# Development and external validation of machine learning approaches for risk prediction of cardiovascular disease in individuals with schizophrenia: a nationwide Swedish and Danish study

**DOI:** 10.1136/bmjment-2025-301964

**Published:** 2026-01-16

**Authors:** Sara Dorthea Nielsen, Maja Dobrosavljevic, Pontus Andell, Zheng Chang, Line Katrine Harder Clemmensen, Henrik Larsson, Michael Eriksen Benros

**Affiliations:** 1 Department of Applied Mathematics and Computer Science, Technical University of Denmark, Lyngby, Denmark; 2 Copenhagen Research Center for Biological and Precision Psychiatry, Mental Health Centre Copenhagen, Copenhagen University Hospital Rigshospitalet, Copenhagen, Denmark; 3 School of Medical Sciences, Faculty of Medicine and Health, Örebro University, Örebro, Sweden; 4 Department of Physiology and Pharmacology, Karolinska Institutet, Stockholm, Sweden; 5 ME Cardiology, Heart and Vascular Theme, Karolinska University Hospital, Stockholm, Sweden; 6 Department of Medical Epidemiology and Biostatistics, Karolinska Institutet, Stockholm, Sweden; 7 Department of Mathematical Sciences, University of Copenhagen, Copenhagen, Denmark; 8 Department of Clinical Medicine, University of Copenhagen Faculty of Health and Medical Sciences, Copenhagen, Denmark

**Keywords:** Schizophrenia, Schizophrenia Spectrum and Other Psychotic Disorders, Psychiatry

## Abstract

**Background:**

Currently available cardiovascular disease (CVD) risk prediction tools may underestimate the risk in individuals with schizophrenia.

**Objective:**

To develop and externally validate 5-year CVD risk prediction models for people with schizophrenia using large-scale register data in Sweden and Denmark with a machine learning (ML) approach.

**Methods:**

Individuals with a diagnosis of schizophrenia, aged 30 and older and without prior CVD, were followed for up to 5 years. We investigated whether adding additional health-related and socio-demographic predictors to the established CVD risk factors improved predictions and compared ML models with logistic regression. External validation was performed across countries.

**Findings:**

A lasso penalised logistic regression including additional predictors achieved the highest predictive performance, both on Swedish and Danish data, while complex ML models with interaction terms did not provide additional improvements. The area under the receiver operating characteristic curve (AUC) on the internal validation data was 0.745 (95% CI (0.742 to 0.749)) in the Swedish model, and 0.722, 95% CI (0.719 to 0.726) in the Danish model. External validation showed similar performance, yielding an AUC of 0.746, 95% CI (0.741 to 0.751) using the Danish model on the Swedish data, and an AUC of 0.720, 95% CI (0.712 to 0.726) using the Swedish model on the Danish validation data.

**Conclusions:**

Incorporating additional health-related information, such as psychiatric comorbidities and medication use, improved 5-year CVD risk prediction for people with schizophrenia in both countries.

**Clinical implications:**

The models can be deployed between Denmark and Sweden without loss of performance compared with training a model on each country.

**WHAT IS ALREADY KNOWN ON THIS TOPIC**

- People with severe mental illness, such as schizophrenia, are at increased risk of developing cardiovascular diseases (CVD). While there is robust research on CVD risk prediction in the general population, risk prediction models in high-risk psychiatric populations remain understudied.

**WHAT THIS STUDY ADDS**

- This is the first CVD risk prediction model developed in individuals with schizophrenia specifically, with external validation in two countries. We showed that additional CVD risk factors improved the prediction compared with established predictors only, while complex machine learning models did not provide improvements compared with lasso penalised logistic regression.

**HOW THIS MIGHT AFFECT RESEARCH, PRACTICE OR POLICY**

- Our study emphasised the need for clinicians to closely monitor CVD risk in individuals with schizophrenia, as they often face healthcare disparities. Future studies need to further validate the models in non-Nordic countries, investigate their clinical impact and update the models by using more detailed clinical measures of CVD risk factors.

## Introduction

 Schizophrenia spectrum disorders are among the most severe mental health disorders, accompanied by an increased risk for multiple psychiatric and physical health conditions, including cardiovascular diseases (CVDs), and an increased risk of premature death.^1^,^3^ Two-thirds of premature deaths among individuals with schizophrenia spectrum disorders stem from natural causes, with CVDs being the leading cause of death.^3 4^ The aetiology of the increased cardiovascular risk and the related excess mortality in individuals with schizophrenia is multifactorial and includes genetic factors, adverse lifestyle (eg, substance abuse, smoking and diet) and socio-economic factors, as well as adverse treatment effects.^1 5 6^ Individuals with schizophrenia show an increase in multiple cardiovascular risk factors compared with the general population, including higher rates of hypertension, dyslipidaemia, diabetes mellitus and smoking—many of which are also frequently undertreated in individuals with schizophrenia.^1 3 7^ Further, antipsychotic medication can cause adverse metabolic and cardiovascular outcomes.^8^ Conversely, the use of antipsychotic medication in patients with schizophrenia can also have protective effects on cardiovascular health, presumably due to associated healthier lifestyle behaviours and higher adherence to other medication (eg, antihypertensives, statins, antidiabetics) versus non-use of antipsychotic medication.^9^ Additionally, the type of antipsychotics and the use of other psychotropic medication for treatment of psychiatric comorbidities can further increase cardiovascular risk.^10^

To optimise preventive strategies and identify high-risk individuals, cardiovascular risk prediction models have been developed, such as the Framingham Risk Score,^11^ Systematic Coronary Risk Evaluation (SCORE)^12^ and a cardiovascular disease risk algorithm (QRISK).^13^ These risk prediction tools use established cardiovascular risk factors such as blood pressure, blood lipid and glucose profiles, smoking and body mass index. However, these models may underestimate the risk of CVD in people with severe mental illness (SMI), likely due to not considering additional cardiovascular risk factors, including comorbid psychiatric conditions, use of psychotropic medication and adverse socio-demographic factors.^5 14^ Nevertheless, efforts to acknowledge the role of SMI in cardiovascular risk have been made recently. The latest version of the QRISK models, QRISK3,^13^ a 10-year CVD risk prediction model developed in the general population aged 25–84, also includes atypical antipsychotics and diagnosis of SMI among CVD risk predictors. Furthermore, the UK-based prediction and management of cardiovascular risk in people with SMI (PRIMROSE) Research Programme^14^ has shown that models including both established cardiovascular risk factors, as well as additional predictors, performed better compared with the models with only established CVD risk factors. Moreover, recently developed CVD risk prediction models using machine learning (ML) algorithms have shown good predictive performance in the general population,^15^,^17^ and in individuals with mental illness.^18^ However, none of the previous risk prediction models have considered individuals with schizophrenia specifically, in whom the risk for CVD is the highest compared with other psychiatric populations.^7 19^

In the present study, we aimed to develop the first 5-year risk prediction model of CVD in individuals with schizophrenia spectrum disorders by using large-scale data from Swedish and Danish electronic health registers, and ML methods to explore a wide range of potential predictors of CVD and potential interactions between the predictors. We first investigated whether by adding additional risk factors (ie, additional health-related information and socio-demographic predictors), the predictive performance of the model improves compared with using established risk factors only. Second, we investigated whether the predictive performance improved by using ML methods compared with using standard approaches (ie, logistic regression). Finally, we developed models independently in Sweden and Denmark, followed by external validation of developed models (ie, external validation of the Swedish model on Danish data and vice versa).

## Methods

We followed the TRIPOD+AI (Transparent Reporting of a multivariable prediction model for Individual Prognosis or Diagnosis+artificial intelligence) statement: updated guidance for reporting clinical prediction models that use regression or ML methods.^20^ Data management was performed using SAS software V.9.4 and R V.4.3.1, and statistical analyses were conducted using Python V.3.11.9 (Scikit-learn, imblearn and eXtreme Gradient Boosting Machine (XGBoost)). The study protocol is provided in online supplemental material.

### Data sources

We used data from record linkages from multiple Swedish and Danish population-based registers. The description of all used registries is in online supplemental material, page 4.

### Population and study period

The study population consisted of individuals aged ≥30 and born between 1932 and 1984, with at least one inpatient or outpatient diagnosis of schizophrenia spectrum disorders (International Classification of Diseases, Tenth Revision (ICD-10) codes: F20-F29) between 2007 and 2014, without previous CVDs. The inclusion period started on 1 January 2007, to allow for enough time for medication prescriptions to be recorded in the Swedish population (ie, at least 18 months from the start of the Prescribed Drug Register on 1 July 2005), and ended in 2014, to allow for a 5-year follow-up until the end of 2019. We selected the end of 2019 as the end of follow-up instead of the last available date in the used data linkages at the end of 2021, to avoid any confounding due to potential differences in diagnosing patterns during the COVID-19 pandemic. Individuals from the cohort were followed from the date of schizophrenia diagnosis, which was registered after 2007 and after age 30 (ie, first diagnosis after these two dates), until they acquired a diagnosis of CVD, emigrated from Sweden/Denmark, died, or by the end of 5 years. From there, individuals were randomly assigned to either the 80% development data set or the 20% hold-out validation set.

**Figure 1:**
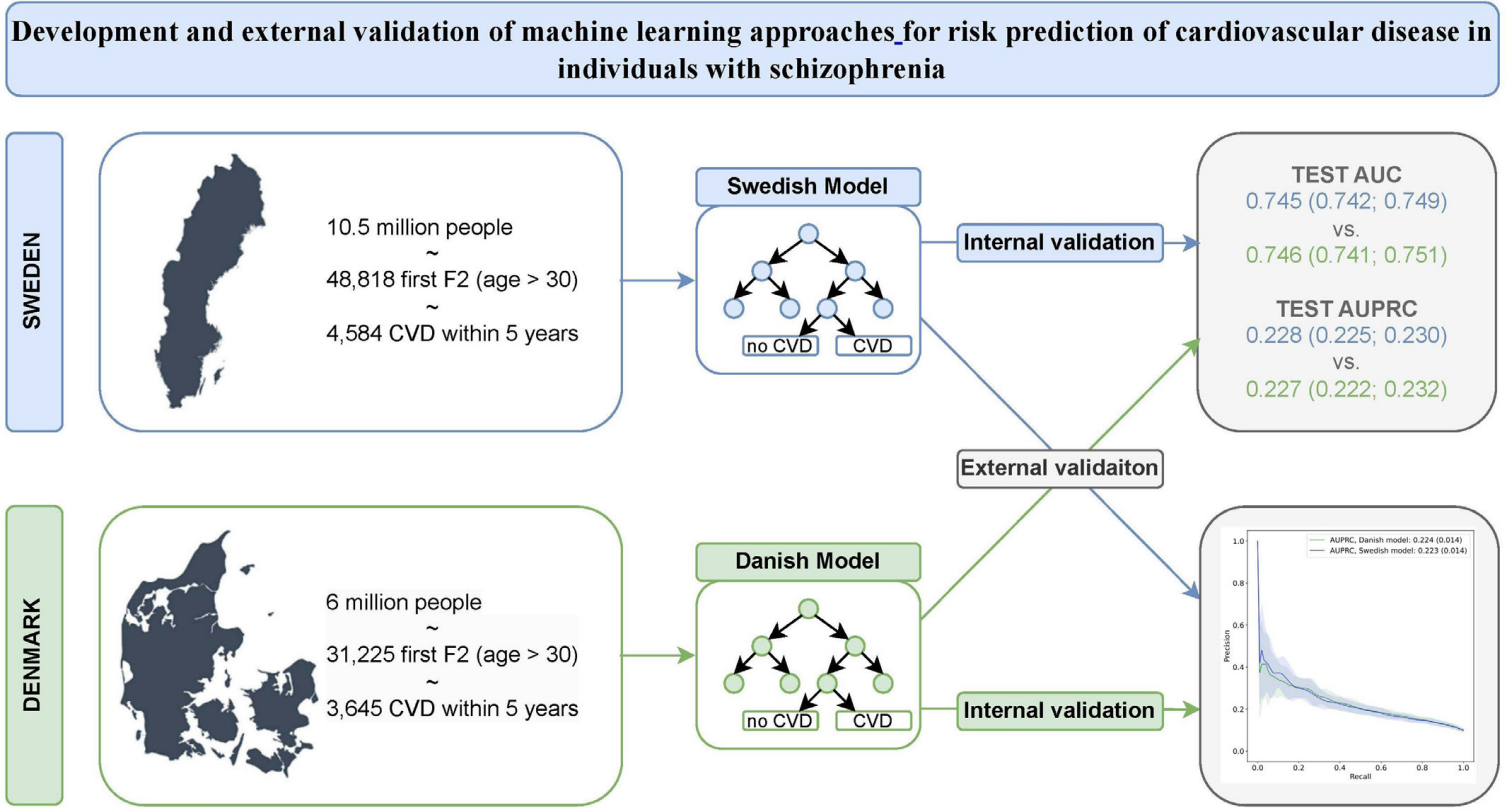
Schematic overview of the study population, study design and structure of data analysis. AUC, area under the receiver operating characteristic curve; AUPRC, area under the precision-recall curve; CVD, cardiovascular disease.

We also created a prospective validation set in the Danish population with an inclusion period between 2015 and 2016 (end of follow-up in end of 2021). The schematic overview of the study population, study design and structure of data analysis is provided in figure 1.

### Candidate predictors

We considered a wide range of potentially relevant cardiovascular risk factors, based on previous literature and established CVD risk prediction models. First, we included proxy measures of established CVD risk factors (ie, blood pressure, lipid status/total/high-density and low-density lipoprotein (LDL) cholesterol, body mass index, smoking and glucose levels),^11^,^13^ which are available in the Swedish and Danish electronic health registers: age, sex, a diagnosis/medication prescription for hypertension, diabetes mellitus (both type I and type II), hyperlipidaemia, obesity and smoking; as well as family history of CVDs.

Second, we considered potentially relevant additional risk factors—additional health-related information and socio-demographic predictors. Among additional health-related predictors, we considered the number of non-psychiatric and psychiatric hospitalisations, psychiatric comorbidity of schizophrenia (eg, sleep disorders, substance use disorder, bipolar disorder, depressive and anxiety disorders) and use of psychotropic medication, which have been previously associated with a cardiovascular risk.^18^,^10^ We also included relevant socio-demographic variables for cardiovascular risk prediction, such as foreign background, family-related factors (eg, being non-married, having no children), rural residential area and low income.^6^

All diagnoses and medication prescriptions were investigated as separate predictors. We included both lifetime and recent history (within the last 2 years) of diagnoses prior to follow-up start, while for medication prescriptions, we only considered dispensed prescriptions within the last 2 years prior to start of follow-up. For socio-demographic variables, we included the most recent information available prior to the start of follow-up. In total, 76 candidate predictors were investigated, as well as potential interactions between candidate predictors. The complete list of predictors and their definitions is in online supplemental tables 1–3. Categorical predictors were one-hot-encoded and numerical predictors were standardised. We used a separate category for missing data, since we could not assume that the data was missing at random (eg, missing family history data due to being born in a foreign country). We measured variable importance as mean absolute SHapley Additive exPlanations (SHAP) values.^21^

### Outcomes

We considered an incident, primary or any secondary, diagnosis or dispensed treatment prescription for the following CVDs: ischaemic heart disease, cerebrovascular diseases and transient ischaemic attack, thromboembolic diseases, heart failure, arteriosclerosis and arrhythmia, acquired after the start of follow-up. Incident CVDs were defined based on the ICD-10 diagnostic codes from the National Patient Register, covering only specialist care or corresponding dispensed medication prescriptions for the treatment of CVDs based on the Anatomical Therapeutic Classification system codes from the Prescribed Drug Register, covering medication prescription from both primary and specialist care (online supplemental table 4).

### Model development and model selection

Individuals from the total cohort were randomly assigned to either the 80% development data set or 20% hold-out validation set. We stratified the splits by the classes in the outcome variables. To assess the association between CVDs and candidate predictors separately in the Danish and the Swedish population, we first performed logistic regression with only established risk factors and compared it to lasso penalised logistic regression and XGBoost^22^ containing a wider set of predictors (76 predictors). For penalised logistic regression and XGBoost, we applied ten-times repeated (to ensure stability) outcome-stratified cross-validation (CV) with fivefolds to tune hyperparameters. The one SE rule was applied to emphasise simpler models and reduce potential overfitting.^23^ The models were retrained on the entire training data with the optimal configuration. The area under the precision-recall curve (AUPRC) was used as a performance measure for hyperparameter tuning. For each measure, bootstrapping with 200 bootstrap samples was applied to the resulting validation samples to produce 95% CIs with the percentile method.

### Model validation

For internal validation of the models, we used 20% hold-out data sets (stratified by outcome). The performance of external validation was evaluated between the two countries on the same 20% hold-out data set that were used for internal validation. Furthermore, the Danish model was tested on data with schizophrenia diagnoses from 2015 to 2017 (end of follow-up in 2021) to assess how the model performs prospectively in time. We used bootstrapping for the internal, external and prospective validation with 200 bootstrap samples to achieve 95% percentile CIs for the performance measures. To assess the discrimination of the model, the area under the receiver operating characteristic curve (AUC) and the AUPRC were used. To assess the calibration of the model, we used the Brier score^24^ and calibration plots.^25^ Sensitivity, specificity, positive predictive value (PPV) and negative predictive value (NPV) were analysed across a range of prespecified cut-off values based on three predefined high-risk thresholds of predicted probability, including 7.5%,^12 15^ 10%^12 26^ and 20%.^11 13 14 27^ We also provided a 5-year absolute and relative risk for CVD separately for individuals classified as high and low risk according to these risk thresholds. We further assessed the performance of the models across the following subgroups: males and females, and aged younger than 50 and 50 and older.

## Results

The total cohorts of patients with schizophrenia consisted of 48 818 individuals in Sweden, and 31 225 individuals in Denmark, with age range from 30 to 83 years old (figure 1). In the Swedish cohort, 39 056 individuals were randomly assigned to the 80% training set, with 3668 (9.39%) of them developing CVDs, and 9762 individuals were assigned to the 20% validation set, and 916 (9.38%) of them developed a CVD during the 5-year follow-up (online supplemental table 6). In the Danish cohort, 24 980 persons were randomly assigned to the 80% training set, among them 2425 (9.71%) individuals developed CVDs, and there were 6245 individuals who were assigned to the 20% validation set, with 609 (9.75%) of them developing a CVD. Furthermore, the temporal validation set in the Danish cohort consisted of 6244 individuals, among which 611 (9.96%) developed CVD. The prevalence of each predictor for the different derivation samples is specified in online supplemental table 5. Most variables have similar prevalence in Sweden and Denmark with some minor differences. For instance, the frequencies of medication prescriptions for sleep disorder and anxiolytics, and diagnosis of anxiety were higher in Sweden compared with Denmark. Frequencies of prescribed antiepileptics, antidepressants and medication for addictive disorders, and diagnosis of personality disorder and alcohol use disorder were higher in Denmark compared with Sweden.

### Model selection

The training and test performances in the CV of the logistic regression model with established risk factors only, and lasso-penalised logistic regression, and XGBoost models with all available predictors are in table 1. The optimal hyperparameters and searched ranges are in online supplemental table 7.

**Table 1:** Performance in the training and test data sets from the fivefold CV with the optimal hyperparameters in the Swedish and Danish data, as the AUPRC and AUC with 95% CIs

	(A) Logistic regression: established risk factors	(B) Penalised logistic regression: all predictors	(C) XGBoost: all predictors
Swedish data			
Training AUPRC	0.212 (0.210 to 0.215)	0.231 (0.229 to 0.233)	0.226 (0.223 to 0.228)
Test AUPRC	0.213 (0.211 to 0.215)	**0.229 (0.226 to 0.231)**	0.222 (0.219 to 0.224)
Training AUC	0.731 (0.729 to 0.733)	0.749 (0.747 to 0.750)	0.735 (0.731 to 0.738)
Test AUC	0.730 (0.728 to 0.733)	**0.748 (0.742 to 0.752)**	0.734 (0.730 to 0.739)
Danish data			
Training AUPRC	0.194 (0.192 to 0.195)	0.222 (0.219 to 0.223)	0.217 (0.213 to 0.224)
Test AUPRC	0.194 (0.191 to 0.196)	**0.221 (0.218 to 0.223)**	0.213 (0.210 to 0.215)
Training AUC	0.685 (0.684 to 0.690)	0.720 (0.718 to 0.222)	0.707 (0.703 to 0.712)
Test AUC	0.684 (0.683 to 0.689)	**0.721 (0.218 to 0.223)**	0.704 (0.700 to 0.707)

The best performance for each country is highlighted in bold.

AUC, area under the receiver operating characteristic curve; AUPRC, area under precision-recall curve.

For both the Swedish and the Danish data, the lasso-penalised logistic regression with all available predictors (76 predictors) yielded a higher performance than logistic regression with only the established risk factors, while the more complex model (XGBoost) did not increase predictive performance (table 1). Based on non-overlapping CIs concerning both AUPRC and AUC, lasso penalised logistic regression was thus selected for both Swedish and the Danish data and all subsequent results in model evaluation were based on this model. The variables and corresponding parameter estimates are in online supplemental table 8.

### Model evaluation

#### Internal validation

The AUC and AUPRC on the internal validation sets were 0.745, 95% CI (0.742 to 0.749) and 0.228, 95% CI (0.225 to 0.230), respectively, for the Swedish model, and 0.722, 95% CI (0.719 to 0.726) and 0.224, 95% CI (0.221 to 0.227) for the Danish model (table 2). Both models showed good calibration (the AUC and AUPRC curves, and calibration plots are in online supplemental figures 1–3). At a high-risk threshold at 10%, the Swedish model resulted in a PPV of 19.0%, NPV 95.5%, sensitivity 67.8% and specificity of 70.2%. This means that 67.8% of the subsequent CVD events within 5 years of receiving a schizophrenia diagnosis would be successfully predicted with the model, and that 19.0% of the ones identified to be of high risk would indeed experience a CVD event within 5 years. Other performance measures at 7.5%, 10% and 20% sensitivity levels are specified in table 2. The temporal validation on the Danish data yielded an AUC of 0.722, 95% CI (0.718 to 0.726) and an AUPRC of 0.220, 95% CI (0.217 to 0.223). Additional performance measures are available in online supplemental tables 9–10 and online supplemental figures 1–6.

**Table 2:** Performance measures on the internal validation data sets for Sweden (A) and Denmark (B), as the AUPRC and AUC and for prespecified high-risk thresholds at 7.5%, 10% and 20%, with 95% CIs

(A) Sweden						
AUC	0.745 CI (0.742 to 0.749)					
AUPRC	0.228 (0.225 to 0.230)					
Risk threshold	7.5%		10%		20%	
Risk group	High	Low	High	Low	High	Low
N total	777	8985	1016	8746	1982	7780
N with the outcome (%)	206 (26.5)	710 (7.9)	257 (25.3)	659 (7.0)	424 (21.4)	492 (6.3)
5-year event rate (absolute risk) with 95% CI	0.265 (0.235 to 0.297)	0.079 (0.074 to 0.085)	0.253 (0.227 to 0.281)	0.075 (0.070 to 0.081)	0.214 (0.196 to 0.233)	0.063 (0.058 to 0.069)
OR with 95% CI unadjusted	4.20 (3.52 to 5.02)		4.16 (3.53 to 4.89)		4.03 (3.50 to 4.64)	
OR with 95% CI adjusted for sex and age	0.86 (0.68 to 1.10)		0.88 (0.70 to 1.11)		0.93 (0.73 to 1.19)	
Sensitivity	0.775 (0.771 to 0.779)		0.678 (0.675 to 0.682)		0.295 (0.290 to 0.298)	
Specificity	0.586 (0.583 to 0.589)		0.702 (0.769 to 0.704)		0.914 (0.910 to 0.919)	
Positive predictive value	0.162 (0.159 to 0.164)		0.190 (0.188 to 0.194)		0.262 (0.258 to 0.265)	
Negative predictive value	0.962 (0.961 to 0.964)		0.955 (0.954 to 0.955)		0.926 (0.924 to 0.927)	
True positives	709		620		270	
True negatives	5177		6200		8076	
False positives	3658		2635		759	
False negatives	206		295		645	

(B) Denmark						
AUC	0.722 (0.719 to 0.726)					
AUPRC	0.224 (0.221 to 0.227)					
Risk threshold	7.5%		10%		20%	
Risk group	High	Low	High	Low	High	Low
N total	469	5776	625	5620	1249	4996
N with the outcome (%)	141 (30.1)	468 (8.1)	169 (27.0)	440 (7.8)	274 (21.9)	335 (6.7)
5-year event rate (absolute risk) with 95% CI	0.301 (0.261 to 344)	0.081 (0.074 to 0.088)	0.270 (0.237 to 0.307)	0.078 (0.072 to 0.086)	0.219 (0.197 to 0.243)	0.067 (0.060 to 0.074)
OR with 95% CI unadjusted	4.36 (3.60 to 5.16)		4.21 (3.67 to 4.98)		4.07 (3.57 to 4.69)	
OR with 95% CI adjusted for sex and age	0.88 (0.71 to 1.12)		0.91 (0.75 to 1.14)		0.94 (0.75 to 1.20)	
Sensitivity	0.739 (0.737 to 0.742)		0.608 (0.605 to 0.611)		0.243 (0.239 to 0.245)	
Specificity	0.581 (0.579 to 0.584)		0.696 (0.694 to 0.697)		0.939 (0.936 to 0.942)	
Positive predictive value	0.160 (0.157 to 0.164)		0.178 (0.174 to 0.182)		0.302 (0.297 to 0.305)	
Negative predictive value	0.954 (0.950 to 0.957)		0.943 (0.940 to 0.947)		0.920 (0.917 to 0.922)	
True positives	450		370		148	
True negatives	3274		3922		5294	
False positives	2362		1714		342	
False negatives	159		239		461	

AUC, area under the receiver operating characteristic curve; AUPRC, area under precision-recall curve; OR, Odds ratio.

#### Predictor importance

The coefficients of individual predictors are depicted in online supplemental table 8 and the top 20 SHAP values are depicted in online supplemental figure 7. The established risk factors all had non-zero coefficients. Age at start of follow-up was the most predictive variable, while hypertension, diabetes mellitus, obesity and family history of CVD were among the top 20 variables with highest (absolute) SHAP values. Additional risk factors among the 20 highest SHAP values, in both the Danish and the Swedish model, included diagnoses of alcohol use disorder and substance use disorder. Considering important prescribed medications were mood stabilisers, antiepileptic, antipsychotics and antidepressants. Socio-demographic variables such as income, civil status and having children also appeared among the most important predictors in models from both countries.

#### External validation

Testing the Danish model on the Swedish validation set yielded an AUC of 0.746, 95% CI (0.741 to 0.751) and an AUPRC of 0.227, 95% CI (0.222 to 0.232). These results are very similar to the internal validation results from Sweden with overlapping CIs. When testing the Swedish model on the Danish validation data, we saw similar results with an AUC of 0.720, 95% CI (0.712 to 0.726) and an AUPRC of 0.223, 95% CI (0.217 to 0.229). Further performance measures from the two external validations are available in online supplemental table 11 and online supplemental figures 8–10.

#### Sensitivity analysis

The models performed similarly across men and women in both countries, and in the age group analysis in Swedish data, based on overlapping CIs. However, in individuals aged 50 and younger, the AUPRC was 0.215, 95% CI (0.209 to 0.220) compared with 0.230, 95% CI (0.223 to 0.234) for those older than 50 in the Danish data. Moreover, the PPV was higher for the individuals aged 50 or older, while the sensitivity was higher for individuals younger than 50 years of age. Performance measures are in online supplemental tables 12 and 13 and online supplemental figure 11.

## Discussion

This is the first study to develop 5-year prediction models for CVD specialised for people with schizophrenia, by using nationwide registry-based data from Sweden and Denmark, with internal and external validation between these countries. We found that CVD risk prediction significantly improved with additional risk factors compared with using only well-established predictors. In the best performing model with additional predictors, the lasso penalised logistic regression, we achieved sensitivity of 67.8% and PPV of 19.0% in the Swedish hold-out validation set, and sensitivity of 60.8% and PPV of 17.8% in the Danish hold-out validation set at a 10% threshold. These results outperform previous studies reporting these metrics.^14 15^

It is plausible that the use of binary, proxy measures instead of more detailed clinical data for established risk factors can, to a certain extent, explain the identified improvement in predictive performance after adding additional health-related and socio-demographic factors. Indeed, CVD risk prediction models using ML and more detailed data (ie, sex, age, LDL, systolic blood pressure, smoking) have not identified such improvements with additional information on psychiatric comorbidity.^18^ Furthermore, more complex ML models, such as XGBoost, which allow for complicated interactions, did not improve the performance compared with the lasso penalised logistic regression without interaction terms. This finding is in line with some previous studies investigating the prediction of chronic diseases with low incidence (eg, CVDs, diabetes, hypertension), which have also identified an overall lack of interactions among predictors and the lack of strong non-linear associations between predictors and outcomes.^28^

We confirmed the relevance of all established CVD risk factors.^11^,^1318^ Among these, old age, hypertension, diabetes and obesity were among the most important predictors in both the Swedish and the Danish model. Several other diagnoses were important in both the Danish and the Swedish model, including diagnosis of alcohol use disorder and substance use disorder. These findings confirm previous research that has identified an even more pronounced risk of CVD in people with SMI and substance abuse compared with those with SMI but without comorbid substance abuse.^29^ Important prescribed medication in both models included antiepileptic, antipsychotics, anxiolytics, antidepressants and smoking cessation medication. These results support previous findings indicating increased cardiovascular risk associated with psychotropic medication or with psychiatric conditions which are treated with these medications (ie, anxiety and depression).^8 10^ We also found that some of the socio-demographic predictors which may be inversely associated with increasing age, for instance, having single status and having low income, may have a protective role in CVD risk prediction in this population. Furthermore, differences in the predictor relevance between the two countries might be due to the difference in the prevalence and medication prescription practices between them.

We found that the Swedish model performed well when externally validated in Denmark, and vice versa. However, we saw a slightly higher AUC and AUPRC on the Swedish validation set compared with the Danish validation set—for both the Danish and Swedish model. Thus, the performance differs slightly depending on the data on which the model is validated and not the data on which the model is trained. This might be due to differences in the prevalence of certain risk factors or clinical practices between Sweden and Denmark. However, we can expect a similar performance within a country independently of whether the model is trained in Sweden or Denmark.

Furthermore, the models performed equally well across men and women in both countries. However, predictive performance for individuals aged older than 50 was different compared with patients aged 50 and younger in the Danish data, with the similar pattern of results in the external validation. The PPV was higher for individuals aged 50 and older compared with those younger than 50, while the sensitivity was higher for individuals younger than 50 years old. Thus, it is important to be aware of the difference in predictive performance between demographic groups before potentially deploying ML models in clinical practice.

Regarding potential clinical implications of the derived models, the target population and clinical context is individuals with schizophrenia aged 30 and older, during a single psychiatric visit. As the models combine lifetime and recent history of diagnoses and medication prescriptions, they could also be used during follow-up psychiatric visits, but this would entail their further evaluation by using such data. Additionally, more detailed clinical data may be needed to update the models and provide adequate tools for repeated risk assessment.

Our study emphasised that clinicians need to be aware of and closely monitor not only the established CVD risk factors in this population, but also to monitor psychiatric comorbidities, psychotropic medication use and socio-economic status, to provide adequate CVD risk prediction and management. Importantly, variables related to socio-demographic status included in the models should be interpreted with caution, as they may partly reflect structural disadvantages and inequalities in access to prevention and care rather than individual biological risk. Although additional risk factors provide only modest increment in the predictive performance on top of the established predictors, this increment may be clinically relevant in high-risk groups such as this. Nevertheless, subgroup-specific performance evaluation and ongoing monitoring of model behaviour across socio-demographic groups would be essential prior to clinical implementation, particularly in settings outside Nordic universal healthcare systems.

### Strengths and limitations

This is the first study to develop CVD risk prediction models by using nationwide population-based data in individuals with schizophrenia in Sweden and Denmark, and which also performed external validation of the derived models between the two countries. This is a pivotal strength, as many existing prediction models of health outcomes lack external validation. Nevertheless, future studies investigating the clinical impact of the derived models are the crucial next step. The derived models showed good discrimination and calibration, and they confirmed the relevance of both established and additional risk factors by using register-based data.

Limitations of the present study may include information bias—used register-based data are only proxy measures (ie, clinical diagnosis/medication prescriptions) of commonly used detailed cardiometabolic risk indicators (eg, blood pressure, blood glucose and lipid levels, body mass index) and health-related behaviours (smoking). Thus, we might have captured only the most severe clinical presentations of these risk factors. This is particularly relevant in individuals with schizophrenia who may experience inequalities regarding CVD risk management and potential underdiagnosis of the covered risk factors and outcomes compared with the general population.^30^ We also did not have access to more detailed information on some medication prescriptions and other important variables, such as physical activity and nutrition. Additionally, all data are attained retrospectively from health records. Currently available national health records systems in Sweden and Denmark do not allow for an automatic data entry from each visit into risk prediction tools (ie, fully dynamic risk prediction tools). Future studies may need to update the models with detailed clinical measures as well as data from follow-up visits to provide more precise cardiovascular risk prediction, or direct model refinement and integration into the clinical electronic health records system. Furthermore, subsequent studies need to investigate clinically relevant time points for risk-reassessment and model updating following established clinical guidelines. Nevertheless, the use of binary measures, that is, the presence/absence of medical history of diagnoses and medication prescriptions from a single visit may be valuable in simplifying the process of collecting information on predictors. This can be relevant in psychiatric healthcare, as well as settings with limited clinical resources for obtaining more detailed data (eg, biochemical measures) and without a possibility for an automatic updating of the risk prediction at each visit. Future studies should also compare the performance of the derived models to models trained on the general population to further establish the need for population-specific models. Finally, although the models trained on Swedish data were externally validated by using truly independent samples from Denmark, and vice versa, the considered countries still have similar socio-demographic conditions and healthcare systems. Thus, the current model may be limited to specialist care of individuals with a diagnosis of schizophrenia spectrum disorders in these countries. To address these potential issues with selection bias, further external validations of the models in non-Nordic countries and across different healthcare systems are warranted.

## Conclusion

We developed and externally validated 5-year CVD risk prediction models in individuals with schizophrenia by using nationwide registry data in Sweden and Denmark. We found that by adding additional health-related variables and socio-demographic factors, the predictive performance of the models improved compared with only using established risk factors. Furthermore, we found that more complex ML models did not improve the performance compared with penalised logistic regression. Both models developed in Sweden and Denmark performed equally well when externally validated in the other country. Future studies are needed to further validate the models in other, non-Nordic countries, to investigate their clinical impact and to update the models by using more detailed clinical measures of CVD risk factors.

## Supplementary material

10.1136/bmjment-2025-301964 online supplemental file 1

## Data Availability

No data are available.
