# Supplementary material for: Development and external validation of machine learning approaches for risk prediction of cardiovascular disease in individuals with schizophrenia: a nationwide Swedish and Danish study
Source: BMJ Ment Health. 2026 Jan 16;29(1):e301964. doi: 10.1136/bmjment-2025-301964 (PMC12815116; doi:10.1136/bmjment-2025-301964)

**Supplementary material**

**Development and external validation of risk prediction models for cardiovascular disease in individuals with schizophrenia using Swedish and Danish population-based register data and machine learning**

**Study protocol *Page 3***

**Data sources *Page 4***

**Supplementary Table 1.** Broad set of candidate predictors of cardiovascular disease in people with psychiatric disorders for machine learning models ***Page 5***

**Supplementary Table 2.** ICD-8/9/10 for health-related variables and ATC codes for corresponding medication prescriptions ***Page 7***

**Supplementary Table 3.** Groups of medication prescriptions with ATC codes ***Page 9***

**Supplementary Table 4.** List of cardiovascular diseases considered as outcomes (ICD-10) and predictors (1st-degree family history, ICD-8/9/10) and corresponding ICD codes ***Page 10***

**Supplementary Table 5.** Baseline characteristics of the study populations regarding the candidate predictors in the derivation and testing data sets ***Page 11***

**Supplementary Table 6.** Characteristics of the study population in the derivation and testing data sets regarding cardiovascular outcomes. ***Page 19***

**Supplementary Table 7.** Hyperparameter ranges and optimal hyperparameters. ***Page 20***

**Supplementary Table 8.** Variables and corresponding parameter estimates in lasso penalized logistic regression. ***Page 21***

**Supplementary Table 9.** Additional performance measures on the internal hold-out test sets for Sweden (A) and Denmark (B) for risk thresholds at 7.5%, 10%, and 20%. ***Page 24***

**Supplementary Table 10.** Performance measures on the temporal hold-out test set on the Danish data for risk thresholds at 7.5%, 10%, and 20%. ***Page 24***

**Supplementary Table 11.** Additional performance measures on external validation for Sweden (A) and Denmark (B) for risk thresholds at 7.5%, 10%, and 20%. ***Page 25***

**Supplementary Table 12.** Performance measures on the internal hold-out test sets for Sweden (A) and Denmark (B) for risk thresholds at 10% separated by sex. ***Page 26***

**Supplementary Table 13.** Performance measures on the internal hold-out test sets for Sweden (A) and Denmark (B) for risk thresholds at 10% separated by age. ***Page 27***

**Supplementary Figure 1.** Internal test AUC curve for Danish (A) and Swedish (B) cohort. ***Page 29***

**Supplementary Figure 2.** Internal test AUPRC curve for Danish (A) and Swedish (B) cohort. ***Page 30***

**Supplementary Figure 3.** Internal test calibration plot for Danish (A) and Swedish (B) cohort. ***Page 31***

**Supplementary Figure 4.** Test AUC curve for temporal hold-out test set Danish cohort. ***Page 32***

**Supplementary Figure 5.** Test AUPRC curve for temporal hold-out test set Danish cohort. ***Page 32***

**Supplementary Figure 6.** Test calibration plot for temporal hold-out test set Danish cohort. ***Page 33***

**Supplementary Figure 7.** Mean SHAP values for Swedish (A) and Danish (B) cohort. ***Page 34***

**Supplementary Figure 8.** External test AUC curve for Swedish (A) and Danish (B) cohort. ***Page 36***

**Supplementary Figure 9.** External test AUPRC curve for Swedish (A) and Danish (B) cohort. ***Page 37***

**Supplementary Figure 10.** External test calibration plot for Swedish (A) and Danish (B) cohort. ***Page 38***

**Supplementary Figure 11.** Internal test calibration plot for Swedish (A) and Danish (B) cohort separated by age. ***Page 39***

**Study protocol**

**Background**

Schizophrenia is a chronic and severe mental health illness with an increased risk of cardiovascular diseases (CVDs), and consequently, premature mortality and shorter life expectancy due to CVD. In order to create individualized treatment and prevention plans for CVD, it is crucial to identify individuals who are at high risk. However, traditional prediction models of CVD, such as Framingham Risk Score, may underestimate the risk in the population with schizophrenia. In the current study, we plan to develop a risk prediction model of incident CVD in people diagnosed with schizophrenia spectrum disorders by combining traditional/established risk factors of CVD (e.g., sex, age, type 2 diabetes, hyper/dyslipidemia, hypertension, obesity, smoking, family history of CVD), with potentially relevant novel predictors associated with schizophrenia and an increased risk of CVDs (i.e., comorbidities and socio-demographic variables) and by using data from Swedish and Danish population-based registries. The considered candidate predictors were selected based on previous literature/expert opinion, data quality and a potential for generalizability to other countries and contexts.

**Research question(s):**

- Do novel risk factors (i.e., physical, and psychiatric comorbidity, use of psychotropic medication, and socio-demographic variables) of CVD improve the predictive accuracy of CVDs in individuals with schizophrenia compared to using traditional cardiovascular risk factors only (i.e., sex, age, type 2 diabetes, hyper/dyslipidemia, hypertension, obesity, smoking, family history of CVD)? The full list of predictors is available in Supplementary Table 1.

- Does ML improve predictions compared to standard methods (logistic regression model)?

- Does sampling improve fairness and generalizability?

- How does the model(s) generalize to temporal test set?

- How does model generalize to other population (Denmark vs. Sweden)?

**Population:** Individuals aged ≥ 30 and born between 1932 and 1988, who were diagnosed with schizophrenia spectrum disorders between January 1st, 2007, and December 31, 2018, and without previous history of CVDs.

**Outcome:** Incident diagnosis of CVD (i.e., primary or any secondary diagnosis) according to the ICD-10 codes, or dispensed medication prescription based on ATC codes (ICD and ATC codes are provided in Table 4 in the appendix), of the following: ischemic heart disease, cerebrovascular disease, venous thrombo-embolism, arteriosclerosis, heart failure, and arrhythmias.

**Time period / follow-up:** We will use a 5-year follow-up after the date of a diagnosis of schizophrenia, established between January 1st 2007, and December 31st of 2014.

**Planned analyses:** We will apply logistic regression, Lasso penalized logistic regression, and Explainable Boosting Machine to assess the association between CVDs and candidate predictors separately in the Danish and the Swedish population. Cross validation will be used for interval validation of the models. For penalized logistic regression and explainable boosting machine, we will apply nested cross validation with five folds to tune hyperparameters in the inner folds and assess performance in the outer folds. The models will be retrained in the outer loop with the optimal configuration. Average precision will be used as performance measure for hyperparameter tuning. We will compare the performance of the standard cox regression and the more advanced machine learning model. External validation will be performed between the two countries. Furthermore, the Danish model will be tested on data from 2019-2021 to assess how the model will perform prospective in time. We will use bootstrapping for both the external test sets and the prospective test set with 200 bootstrap samples to achieve confidence intervals. We will sample the training data to improve predictions for underrepresented observations using inverse density sampling. To assess the discrimination of the model, the AUROC and AUPRC will be used. To assess the calibration of the model we will use the Brier score and calibration plots. We will also compare the performance of the model that includes only traditional risk factors with the model which includes additional non-traditional risk factors by calculating the AUROC and AUPRC.

**Data sources**

We used data from record linkages from multiple Swedish and Danish population-based registers. All diagnoses were obtained from the National Patient Registers (NPR) and the Cause of Death Registers (CDR). The Swedish NPR contains information on all in-patient diagnoses since 1987, and out-patient diagnoses since 2001 (1), while Danish NPR contains information on in-patient diagnoses since 1977, and outpatient diagnoses since 1995 (2). The Swedish CDR contains information on all deaths since 1952 (3), and Danish since 1875 (4). In Denmark, psychiatric diagnoses prior to 1995 were obtained from the Danish Psychiatric Central Research Register (PCR) (5). All diagnoses in the NPR, PCR, and CDR were classified according to the International Classification of Diseases (ICD) (diagnostic codes are in the Supplementary Table 2-4). The Swedish Prescribed Drug Register (PDR), dating from July 2005 (6), and the Danish PDR, dating from 1994 (7), cover data on all dispensed medication prescriptions using the Anatomical Therapeutic Classification (ATC) system, with a date of prescription and dosage.

Socio-demographic data were obtained from the Total Population Register (TPR) (8) in Sweden and the Danish Civil Registration System Register (9) in Denmark, which contain demographic information from 1968. In Sweden, the Longitudinal integration database for health insurance and labour market studies register (LISA) contains relevant information on income, civil status, etc., since 1990 on all individuals aged 16 and older (10), while the Multigeneration-generation Register (11) was used to link study population with their first-degree relatives and acquire information on medical family history from the NPR. In Denmark, disposable income is available in the Danish Income Register (12) since 1980 on individuals aged 15 or older, while the Medical Birth Register (13) was used to link the study population with their first-degree relatives.

References:

1. Ludvigsson JF, Andersson E, Ekbom A, Feychting M, Kim JL, Reuterwall C, et al. External review and validation of the Swedish national inpatient register. BMC Public Health. 2011;11.

2. Lynge E, Sandegaard JL, Rebolj M. The Danish national patient register. Scand J Public Health. 2011 Jul;39(7):30–3.

3. Brooke HL, Talbäck M, Hörnblad J, Johansson LA, Ludvigsson JF, Druid H, et al. The Swedish cause of death register. Eur J Epidemiol. 2017 Sep 1;32(9):765–73.

4. Sundhedsstyrelsen (2010) Registerdekleration for Dødsårsagsregistret. Available from [www.sst.dk/Indberetning%20og%20statistik/Doedsaarsagsregisteret.aspx](http://www.sst.dk/Indberetning%20og%20statistik/Doedsaarsagsregisteret.aspx), accessed 20 October 2010.

5. Mors O, Perto GP, Mortensen PB. The Danish psychiatric central research register. Scand J Public Health. 2011 Jul;39(7):54–7.

6. Wettermark B, Hammar N, Fored CM, Leimanis A, Olausson PO, Bergman U, et al. The new Swedish Prescribed Drug Register Opportunities for pharmacoepidemiological research and experience from the first six months. Pharmacoepidemiol Drug Saf. 2007;16(7):726–35.

7. Wallach Kildemoes H, Toft Sørensen H, Hallas J. The Danish national prescription registry. Scand J Public Health. 2011 Jul;39(7):38–41.

8. Ludvigsson JF, Almqvist C, Bonamy AKE, Ljung R, Michaëlsson K, Neovius M, et al. Registers of the Swedish total population and their use in medical research. Eur J Epidemiol. 2016 Feb 1;31(2):125–36.

9. Pedersen CB. The Danish civil registration system. Scand J Public Health. 2011;39(7_suppl):22–5.

10. Ludvigsson JF, Svedberg P, Olén O, Bruze G, Neovius M. The longitudinal integrated database for health insurance and labour market studies (LISA) and its use in medical research. Eur J Epidemiol. 2019 Apr 1;34(4):423–37.

11. Ekbom A. The Swedish Multi-generation Register. In: Methods in Molecular Biology. Humana Press Inc.; 2011. p. 215–20.

12. Baadsgaard M, Quitzau J. Danish registers on personal income and transfer payments. Scand J Public Health. 2011 Jul;39(7):103–5.

13. Bliddal M, Broe A, Pottegård A, Olsen J, Langhoff-Roos J. The Danish Medical Birth Register. Vol. 33, European Journal of Epidemiology. Springer Netherlands; 2018. p. 27–36.

**Supplementary Table 1.** Broad set of candidate predictors of cardiovascular disease in people with psychiatric disorders for machine learning models.

Note: health-related predictors, diagnoses and medications prescriptions are binary variables, only the number of hospitalizations is continuous.

|  | **Health related predictors: diagnoses^1^** | |
| --- | --- | --- |
| 1 | Hypertension | |
| 2 | Type 1 diabetes | |
| 3 | Type 2 diabetes | |
| 4 | Hyperlipidemia | |
| 5 | Obesity | |
| 6 | Tobacco use disorder | |
| 7 | Family history of CVD (1^st^ degree relative, by age 60) parents | |
| 8 | Family history of CVD (1^st^ degree relative, by age 60) full siblings | |
| 9 | Death of a parent due to a cause other than CVD before age 60 | |
| 10 | Dementia diagnosis | |
| 11 | Epilepsy | |
| 12 | Migraine | |
| 13 | Arthritis/osteoarthritis/joint pain | |
| 14 | Autoimmune diseases | |
| 15 | Anxiety disorders | |
| 16 | Depressive disorders | |
| 17 | Bipolar disorder | |
| 18 | Attention deficit/hyperactivity disorder | |
| 19 | Substance use disorder other than tobacco and alcohol | |
| 20 | Sleep disorders | |
| 21 | Alcohol use disorder | |
| 22 | Intellectual disability | |
| 23 | Pervasive developmental disorders | |
| 24 | Personality disorders | |
| 25 | Eating disorders | |
| 26 | Family history (1^st^ degree relative) of psychiatric illness | |
| 27 | Number of nonpsychiatric hospitalizations within last 2 years | |
| 28 | Number of psychiatric hospitalizations within last 2 years | |
|  | **Health related predictors: Medication prescriptions^2^** | |
| 1 | Hypertension medication prescription | |
| 2 | Hyperlipidemia medication prescription | |
| 3 | Medication prescription for smoking cessation | |
| 4 | Sleep disorders medication | |
| 5 | Corticosteroids | |
| 6 | Alzheimer’s disease medication prescription | |
| 7 | Anxiolytics | |
| 8 | Hypnotics and sedatives (except medication for sleep disorders) | |
| 9 | Antiepileptics | |
| 10 | Antidepressants | |
| 11 | Mood stabilizers | |
| 12 | 1st generation antipsychotics | |
| 13 | 2nd generation antipsychotics | |
| 14 | Medication used for the treatment of ADHD | |
| 15 | Drugs used for addictive disorders (alcohol and opioid dependance) | |
|  | **Sociodemographic variables^3^** | **Description/definition** |
| 1 | Age at the start of follow-up | -continuous variable |
| 2 | Male sex | -Binary |
| 3 | Educational attainment (2 dummy variables) | -Categorical - 3 levels: <=9 years/10-12 years/>12 years |
| 4 | Income^4^ (4 dummy variables) | -Categorical – 5 levels: Negative (in debt)/Zero/Low (<20th percentile)/Medium (20th – 80th percentile)/ High (>80th percentile) |
| 5 | Birth country | -Binary:  1= Being foreign born (in a country other than Sweden/Denmark)  0= Born in Sweden/Denmark |
| 6 | Area of residence | -Binary  Urban: Large cities and commuting municipalities near large cities; Medium-sized towns and commuting municipalities near medium-sized towns; Smaller towns and commuting municipalities near smaller towns  Rural: Rural municipalities with a population of less than 15 000, very low commuting rate (less than; 30 %); Rural municipalities with a visitor industry. |
| 7 | Civil status | -Binary:  1= Surviving partner/ unmarried/ divorced/divorced partner/widow/er  0= Married/ /registered partner |
| 8 | -Having children | -Binary |
| 9 | -Having children with different partners | -Binary |

^1^There are two time-windows for diagnoses: any history of a diagnosis, and within last 2 years prior to start of follow up

^2^Time window for medication prescriptions is within 2 years prior to follow up start

^3^Time window for SES variables is set at the year with available information closest (prior) to the follow up start.

^4^ Personal disposable income was standardized in accordance with population distribution for the corresponding year

**Supplementary Table 2.** ICD-8/9/10 for health-related variables and ATC codes for corresponding medication prescriptions

|  | **ICD-8** | **ICD-9** | **ICD-10** | **ATC** |
| --- | --- | --- | --- | --- |
| Hypertensive diseases (diagnosis and medication prescription) | 400-404 | 401-405 | I1 | C03A, C08C, C09A, C09B, C09C |
| Type 1 diabetes (diagnosis and medication prescription) | 250 | 250 | E10 | A10 (any) |
| Peripheral artery disease (type 2 diabetes) (diagnosis and medication prescription) | 250 | 250 | E11 |  |
| Obesity (diagnosis) | 277 | 278A, 278B | E65-E66 | - |
| Hyperlipidemia | 279 | 272 | E78 | C10, C10A, C10B |
| Smoking (tobacco use disorder diagnosis and medication for smoking cessation) | _ | - | F17 | N07BA01, N07BA03 |
| Alzheimer’s disease and other dementias diagnosis, and medication used for treatment of Alzheimer’s disease | 290, 2930, 2931 | 290A, 290B, 290E, 290W, 290X, 294B, 331B, 331A, 331C, 331X | G30, G31.1, G31.8, F00-F03, F05.1 | N06D |
| Epilepsy | 345 | 345 | G40-41 | - |
| Migraine | 34609 | 346A/B/X/W | G43 | - |
| Arthritis/osteoarthritis/joint pain (diagnosis) | - | - | M0, M1, M2, R26 | - |
| Autoimmune disease – any (diagnosis) | 0341 13607 24200 24503 25810 26910 28700 28710 340 35401 390 391 392 44609 44630 44638 44640 56300 56310 57190 580 582 694 69610 69619 69620 69621 69622 69623 70400 71200 71210 71239 71600 73300 73400 73410 | 034B 136B 242A 245C 258B 287A 287D 340 357A 358A 390 391 392 446A 446B 446F 446G 555 556 571F 579A 580 582 694A 694E 694F 696 704A 710A 710B 710C 710D 710W 714A 725 | A389 D686 D690 D693 E050 E063 E310 G04 G131 G35 G610 G700 I00 I01 I02 L100 L120 L13 L40 L63 K900 K50 K51 K743 M06 M300 M301 M303 M311 M315 M317 M32 M339 M34 M350 M351 M352 M353 N00 N01 N03 N05 | - |
| Sleep disorders (diagnosis and medication prescription) | 306.40 | 307E, 780F | G47, F51 | N05CH01, N05CF01, N05CF02, N05CF03 |
| Anxiety disorders | 300 (except 300.4) | 300 (except 300E) | F40-F42, F45 |  |
| Depression | 2962.x, 3004.x | 296B.x, 300E.x, 311 | F32.x, F33.x, F34.x, F38 F39 |  |
| Attention-deficit/hyperactivity disorder | _ | 314 | F90 |  |
| Bipolar disorder | 296.0–296.3, 296.8, 296.9 | 296A-296E, 296W, 296X | F30, F31 |  |
| Schizophrenia | 295 except 295.7 | 295A–295E, 295G, 295W, 295X | F20 |  |
| Pervasive developmental disorders | - | 299A.x | F84.x |  |
| Intellectual disability | 310, 3105.x, 311, 312, 313, 314, 315 | 317, 318, 319, 758 | F70, F71, F72, F73, F78, F79, Q9 |  |
| Personality disorders | 301 | 301 | F6x, F60x |  |
| Eating disorders | - | 307B.x, 307F.x | F50 |  |
| Alcohol use disorder | 291.x, 303x | 291.x, 303x, 305A | F10 |  |
| Substance use disorder other than alcohol and tobacco | 304x | 304, 305x (except 305A and 305B), 292x | F11-F19, except F17 |  |

**Supplementary Table 3.** Groups of medication prescriptions with ATC codes

|  | **ATC** |
| --- | --- |
| Corticosteroids | D07, H02 |
| Anxiolytics | N05B |
| Hypnotics and sedatives | N05C (except for medication used for treatment of sleep disorders, see Supplementary table 3) |
| Antiepileptics | N03A, except mood stabilizers |
| Antidepressants | N06A |
| Mood stabilizers | Lithium: N05AN01 |
|  | Other mood stabilizers: N03AG01, N03AX09, N03AF01, N03AF02 |
| Antipsychotics | Typical antipsychotics: N05AA02, N05AB03, N05AD01, N05AD08, N05AF01, N05AF03, N05AF05 |
|  | Atypical antipsychotics: N05AD03, N05AE03, N05AE04, N05AE05, N05AH02, N05AH03, N05AH04, N05AX08, N05AX12, N05AX13 |
| Medication used for the treatment of ADHD | N06BA01, N06BA02, N06BA04, N06BA09, N06BA12, C02AC02 |
| Drugs used for addictive disorders (alcohol and opioid dependance) | N07BB01, N07BB03, N07BB04, N02AE01, N07BC01, N07BC02, N07BC51 |

**Supplementary Table 4.** List of cardiovascular diseases considered as outcomes (ICD-10) and predictors (1st-degree family history, ICD-8/9/10) and corresponding ICD codes*

|  | ICD-8 | ICD-9 | ICD-10 |
| --- | --- | --- | --- |
| Ischemic heart disease | 410-414 | 410-414 | I20, I21, I22, I23, I24, I251, I252, I255, I256, I258, I259 |
| Cerebrovascular diseases and transient ischemic attack | 43 | 43 | G45, I6 |
| Thromboembolic disease | 450, 451 | 415B, 451B | I26, I80 |
| Arteriosclerosis | 440-444 | 440-444 | I70-I74 |
| Heart failure | 428 | 428 | I42, I50 |
| Arrhythmia | - | 426A, 426B, 427A, 427B, 427D, 427E, 427F, 427W | I441, I442, I46, I470, I471, I472, I48, I490, I495, I498 |

*ATC codes used for identifying any CVD: C01A, C01B, C01D, C01E

**Supplementary Table 5.** Baseline characteristics of the study populations regarding the candidate predictors within last two year and any/lifetime history prior to start of follow up.

1. **Swedish Cohort**

|  | **Train** | **Train** | **Train** | **Test** | **Test** | **Test** |
| --- | --- | --- | --- | --- | --- | --- |
|  | **All (N=38,999)** | **With incident CVD (N=3,663)** | **Without incident CVD (N=35,336)** | **All (N=9,750)** | **With incident CVD (N=915)** | **Without incident CVD (N=8,835)** |
| **Age (SD)** | 48.66 (12.26) | 57.78 (11.46) | 47.72 (11.95) | 48.79 (12.23) | 57.53 (11.57) | 47.89 (11.94) |
| **Male sex** | 20302 (52.1%) | 1854 (50.6%) | 18448 (52.2%) | 5148 (52.8%) | 477 (52.1%) | 4671 (52.9%) |
| **Hypertension - last 2 years** | 906 (2.3%) | 231 (6.3%) | 675 (1.9%) | 225 (2.3%) | 61 (6.7%) | 164 (1.9%) |
| **Hypertension – any** | 1728 (4.4%) | 425 (11.6%) | 1303 (3.7%) | 421 (4.3%) | 102 (11.1%) | 319 (3.6%) |
| **Type 1 diabetes - last 2 years** | 413 (1.1%) | 88 (2.4%) | 325 (0.9%) | 93 (1.0%) | 19 (2.1%) | 74 (0.8%) |
| **Type 1 diabetes - any** | 926 (2.4%) | 211 (5.8%) | 715 (2.0%) | 233 (2.4%) | 54 (5.9%) | 179 (2.0%) |
| **Type 2 diabetes - last 2 years** | 941 (2.4%) | 222 (6.1%) | 719 (2.0%) | 233 (2.4%) | 58 (6.3%) | 175 (2.0%) |
| **Type 2 diabetes - any** | 1860 (4.8%) | 399 (10.9%) | 1461 (4.1%) | 451 (4.6%) | 98 (10.7%) | 353 (4.0%) |
| **Hyperlipidemia - last 2 years** | 169 (0.4%) | 29 (0.8%) | 140 (0.4%) | 34 (0.3%) | 5 (0.5%) | 29 (0.3%) |
| **Hyperlipidemia - any** | 382 (1.0%) | 78 (2.1%) | 304 (0.9%) | 74 (0.8%) | 11 (1.2%) | 63 (0.7%) |
| **Obesity – last 2 years** | 356 (0.9%) | 55 (1.5%) | 301 (0.9%) | 78 (0.8%) | 9 (1.0%) | 69 (0.8%) |
| **Obesity - any** | 844 (2.2%) | 129 (3.5%) | 715 (2.0%) | 202 (2.1%) | 26 (2.8%) | 176 (2.0%) |
| **Tobacco use disorder - last 2 years** | 112 (0.3%) | 25 (0.7%) | 87 (0.2%) | 19 (0.2%) | 5 (0.5%) | 14 (0.2%) |
| **Tobacco use disorder - any** | 201 (0.5%) | 40 (1.1%) | 161 (0.5%) | 53 (0.5%) | 11 (1.2%) | 42 (0.5%) |
| **Dementia – last 2 years** | 185 (0.5%) | 48 (1.3%) | 137 (0.4%) | 37 (0.4%) | 6 (0.7%) | 31 (0.4%) |
| **Dementia - any** | 335 (0.9%) | 76 (2.1%) | 259 (0.7%) | 85 (0.9%) | 20 (2.2%) | 65 (0.7%) |
| **Epilepsy – last 2 years** | 524 (1.3%) | 82 (2.2%) | 442 (1.3%) | 122 (1.3%) | 19 (2.1%) | 103 (1.2%) |
| **Epilepsy - any** | 1322 (3.4%) | 195 (5.3%) | 1127 (3.2%) | 311 (3.2%) | 41 (4.5%) | 270 (3.1%) |
| **Migraine - last 2 years** | 84 (0.2%) | 3 (0.1%) | 81 (0.2%) | 29 (0.3%) | < 5 | 25 (0.3%) |
| **Migraine - any** | 342 (0.9%) | 26 (0.7%) | 316 (0.9%) | 109 (1.1%) | 12 (1.3%) | 97 (1.1%) |
| **Arthritis/osteoarthritis/joint pain – last 2 years** | 1178 (3.0%) | 174 (4.8%) | 1004 (2.8%) | 278 (2.9%) | 47 (5.1%) | 231 (2.6%) |
| **Arthritis/osteoarthritis/joint pain - any** | 2894 (7.4%) | 421 (11.5%) | 2473 (7.0%) | 719 (7.4%) | 89 (9.7%) | 630 (7.1%) |
| **Autoimmune disorders – last 2 years** | 860 (2.2%) | 100 (2.7%) | 760 (2.2%) | 215 (2.2%) | 23 (2.5%) | 192 (2.2%) |
| **Autoimmune disorders - any** | 1984 (5.1%) | 231 (6.3%) | 1753 (5.0%) | 528 (5.4%) | 66 (7.2%) | 462 (5.2%) |
| **Anxiety - last 2 years** | 3877 (9.9%) | 330 (9.0%) | 3547 (10.0%) | 983 (10.1%) | 82 (9.0%) | 901 (10.2%) |
| **Anxiety - any** | 8074 (20.7%) | 729 (19.9%) | 7345 (20.8%) | 2033 (20.9%) | 200 (21.9%) | 1833 (20.7%) |
| **Depressive disorders – last 2 years** | 4342 (11.1%) | 381 (10.4%) | 3961 (11.2%) | 1105 (11.3%) | 113 (12.3%) | 992 (11.2%) |
| **Depressive disorders - any** | 10354 (26.5%) | 1006 (27.5%) | 9348 (26.5%) | 2615 (26.8%) | 289 (31.6%) | 2326 (26.3%) |
| **ADHD - last 2 years** | 798 (2.0%) | 57 (1.6%) | 741 (2.1%) | 211 (2.2%) | 11 (1.2%) | 200 (2.3%) |
| **ADHD - any** | 1056 (2.7%) | 69 (1.9%) | 987 (2.8%) | 279 (2.9%) | 14 (1.5%) | 265 (3.0%) |
| **Substance use disorder - last 2 years** | 2527 (6.5%) | 189 (5.2%) | 2338 (6.6%) | 594 (6.1%) | 66 (7.2%) | 528 (6.0%) |
| **Substance use disorder - any** | 6455 (16.6%) | 590 (16.1%) | 5865 (16.6%) | 1572 (16.1%) | 162 (17.7%) | 1410 (16.0%) |
| **Sleep disorders - last 2 years** | 286 (0.7%) | 20 (0.5%) | 266 (0.8%) | 68 (0.7%) | < 5 | 67 (0.8%) |
| **Sleep disorders - any** | 922 (2.4%) | 96 (2.6%) | 826 (2.3%) | 223 (2.3%) | 23 (2.5%) | 200 (2.3%) |
| **Alcohol use disorder – last 2 years** | 2123 (5.4%) | 219 (6.0%) | 1904 (5.4%) | 529 (5.4%) | 72 (7.9%) | 457 (5.2%) |
| **Alcohol use disorder - any** | 5724 (14.7%) | 650 (17.7%) | 5074 (14.4%) | 1471 (15.1%) | 184 (20.1%) | 1287 (14.6%) |
| **Intellectual disability - last 2 years** | 715 (1.8%) | 65 (1.8%) | 650 (1.8%) | 152 (1.6%) | 15 (1.6%) | 137 (1.6%) |
| **Intellectual disability - any** | 1839 (4.7%) | 221 (6.0%) | 1618 (4.6%) | 433 (4.4%) | 39 (4.3%) | 394 (4.5%) |
| **Pervasive developmental disorder – last 2 years** | 640 (1.6%) | 25 (0.7%) | 615 (1.7%) | 168 (1.7%) | 7 (0.8%) | 161 (1.8%) |
| **Pervasive developmental disorder - any** | 983 (2.5%) | 44 (1.2%) | 939 (2.7%) | 253 (2.6%) | 12 (1.3%) | 241 (2.7%) |
| **Schizophrenia – last 2 years** | 7927 (20.3%) | 637 (17.4%) | 7290 (20.6%) | 1987 (20.4%) | 186 (20.3%) | 1801 (20.4%) |
| **Schizophrenia - any** | 14558 (37.3%) | 1439 (39.3%) | 13119 (37.1%) | 3607 (37.0%) | 374 (40.9%) | 3233 (36.6%) |
| **Bipolar disorder – last 2 years** | 1904 (4.9%) | 205 (5.6%) | 1699 (4.8%) | 489 (5.0%) | 43 (4.7%) | 446 (5.0%) |
| **Bipolar disorder - any** | 4664 (12.0%) | 513 (14.0%) | 4151 (11.7%) | 1135 (11.6%) | 116 (12.7%) | 1019 (11.5%) |
| **Personality disorders – last 2 years** | 2196 (5.6%) | 181 (4.9%) | 2015 (5.7%) | 537 (5.5%) | 47 (5.1%) | 490 (5.5%) |
| **Personality disorders - any** | 7482 (19.2%) | 704 (19.2%) | 6778 (19.2%) | 1807 (18.5%) | 184 (20.1%) | 1623 (18.4%) |
| **Eating disorders – last 2 years** | 198 (0.5%) | 8 (0.2%) | 190 (0.5%) | 42 (0.4%) | < 5 | 41 (0.5%) |
| **Eating disorders - any** | 619 (1.6%) | 36 (1.0%) | 583 (1.6%) | 176 (1.8%) | 9 (1.0%) | 167 (1.9%) |
| **Nonpsychiatric hospitalizations – last 2 years** | 0.06 (0.40) | 0.13 (0.63) | 0.05 (0.36) | 0.06 (0.46) | 0.13 (0.61) | 0.05 (0.44) |
| **Psychiatric hospitalizations – last 2 years** | 0.49 (1.74) | 0.50 (1.74) | 0.49 (1.74) | 0.45 (1.58) | 0.56 (1.78) | 0.44 (1.55) |
| **Hypertension medication - last 2 years** | 4139 (10.5%) | 809 (22.1%) | 3321 (9.4%) | 1067 (10.9%) | 210 (23.0%) | 857 (9.7%) |
| **Hyperlipidemia medication - last 2 years** | 2371 (6.1%) | 429 (11.7%) | 1942 (5.5%) | 566 (5.8%) | 92 (10.1%) | 474 (5.4%) |
| **Smoking cessation medication - last 2 years** | 517 (1.3%) | 71 (1.9%) | 446 (1.3%) | 111 (1.1%) | 16 (1.7%) | 95 (1.1%) |
| **Sleep disorders medication - last 2 years** | 12255 (31.4%) | 1219 (33.3%) | 11036 (31.2%) | 3070 (31.5%) | 313 (34.2%) | 2757 (31.2%) |
| **Alzheimer’s disease medication - last 2 years** | 126 (0.3%) | 38 (1.0%) | 88 (0.2%) | 18 (0.2%) | 5 (0.5%) | 13 (0.1%) |
| **Anxiolytics medication - last 2 years** | 14502 (37.2%) | 1522 (41.6%) | 12980 (36.7%) | 3666 (37.6%) | 402 (43.9%) | 3264 (36.9%) |
| **Hypnotics sedatives – last 2 years** | 9782 (25.1%) | 1015 (27.7%) | 8767 (24.8%) | 2405 (24.7%) | 278 (30.4%) | 2127 (24.1%) |
| **Antiepileptics - last 2 years** | 1963 (5.0%) | 234 (6.4%) | 1729 (4.9%) | 465 (4.8%) | 60 (6.6%) | 405 (4.6%) |
| **Antidepressants - last 2 years** | 14557 (37.3%) | 1422 (38.8%) | 13135 (37.2%) | 3679 (37.7%) | 351 (38.4%) | 3328 (37.7%) |
| **Mood stabilizers - last 2 years** | 5595 (14.3%) | 643 (17.6%) | 4952 (14.0%) | 1351 (13.9%) | 146 (16.0%) | 1205 (13.6%) |
| **Typical antipsychotics - last 2 years** | 13099 (33.6%) | 1478 (40.3%) | 11621 (32.9%) | 3284 (33.7%) | 396 (43.3%) | 2888 (32.7%) |
| **Atypical antipsychotics – last 2 years** | 20412 (52.3%) | 1758 (48.0%) | 18654 (52.8%) | 5110 (52.4%) | 439 (48.0%) | 4671 (52.9%) |
| **ADHD medication - last 2 years** | 749 (1.9%) | 53 (1.4%) | 696 (2.0%) | 191 (2.0%) | 11 (1.2%) | 180 (2.0%) |
| **Addictive disorders medication - last 2 years** | 1933 (5.0%) | 181 (4.9%) | 1752 (5.0%) | 442 (4.5%) | 61 (6.7%) | 381 (4.3%) |
| **Income negative** | 204 (0.5%) | 13 (0.4%) | 191 (0.5%) | 40 (0.4%) | < 5 | 38 (0.4%) |
| **Income zero** | 356 (0.9%) | 26 (0.7%) | 330 (0.9%) | 84 (0.9%) | < 5 | 80 (0.9%) |
| **Income low** | 9589 (24.6%) | 794 (21.7%) | 8795 (24.9%) | 2462 (25.3%) | 222 (24.3%) | 2240 (25.4%) |
| **Income medium** | 27480 (70.5%) | 2724 (74.4%) | 24756 (70.1%) | 6852 (70.3%) | 662 (72.3%) | 6190 (70.1%) |
| **Income high** | 1370 (3.5%) | 106 (2.9%) | 1264 (3.6%) | 312 (3.2%) | 25 (2.7%) | 287 (3.2%) |
| **Foreign born** | 9848 (25.3%) | 813 (22.2%) | 9035 (25.6%) | 2453 (25.2%) | 203 (22.2%) | 2250 (25.5%) |
| **Residence rural** | 2443 (6.3%) | 257 (7.0%) | 2186 (6.2%) | 581 (6.0%) | 68 (7.4%) | 513 (5.8%) |
| **Civil status alone** | 32635 (83.7%) | 3019 (82.4%) | 29616 (83.8%) | 8137 (83.5%) | 763 (83.4%) | 7374 (83.5%) |
| **Having children** | 17166 (44.0%) | 1914 (52.3%) | 15252 (43.2%) | 4292 (44.0%) | 475 (51.9%) | 3817 (43.2%) |
| **CVD family history - parent** | 5490 (14.1%) | 396 (10.8%) | 5094 (14.4%) | 1349 (13.8%) | 104 (11.4%) | 1245 (14.1%) |
| **CVD family history - sibling** | 2989 (7.7%) | 452 (12.3%) | 2537 (7.2%) | 722 (7.4%) | 112 (12.2%) | 610 (6.9%) |
| **Family history of psychiatric disorders** | 13540 (34.7%) | 1220 (33.3%) | 12320 (34.9%) | 3418 (35.1%) | 303 (33.1%) | 3115 (35.3%) |
| **Parent died before age 60** | 4695 (12.0%) | 466 (12.7%) | 4229 (12.0%) | 1192 (12.2%) | 120 (13.1%) | 1072 (12.1%) |

1. **Danish Cohort**

|  | **Training data** | | | **Internal test data** | | | **Temporal test data** | | |
| --- | --- | --- | --- | --- | --- | --- | --- | --- | --- |
|  | **All (N=24,980)** | **With incident CVD (N=2,423)** | **Without incident CVD (N=22,557)** | **All (N=6,245)** | **With incident CVD (N=609)** | **Without incident CVD (N=5,636)** | **Total (N=6,409)** | **With incident CVD (N=508)** | **Without incident CVD (N=6917)** |
| **Mean age (SD)** | 46.14 (11.91) | 52.93 (12.66) | 45.41 (11.60) | 45.94 (11.98) | 53.27 (12.98) | 45.14 (11.59) | 43.36 (13.25) | 56.29 (15.56) | 44.31 (13.85) |
| **Male sex** | 14033 (56.2%) | 1328 (54.8%) | 12705 (56.3%) | 3569 (57.2%) | 331 (54.2%) | 3238 (57.5%) | 3588 (56.0%) | 274 (53.9%) | 3862 (55.8%) |
| **Hypertension - last 2 years** | 337 (1.4%) | 83 (3.5%) | 253 (1.1%) | 90 (1.4%) | 27 (4.4%) | 63 (1.1%) | 98 (1.5%) | 32 (6.3%) | 130 (1.9%) |
| **Hypertension – any** | 860 (3.4%) | 199 (8.2%) | 661 (2.9%) | 198 (3.2%) | 59 (9.7%) | 139 (2.5%) | 273 (4.3%) | 66 (13.0%) | 339 (4.9%) |
| **Type 1 diabetes - last 2 years** | 190 (0.8%) | 38 (1.6%) | 152 (0.7%) | 57 (0.9%) | 12 (2.0%) | 45 (0.8%) | 29 (0.5%) | 9 (1.8%) | 38 (0.5%) |
| **Type 1 diabetes - any** | 402 (1.6%) | 89 (3.7%) | 313 (1.4%) | 108 (1.7%) | 26 (4.3%) | 82 (1.5%) | 63 (1.0%) | 14 (2.8%) | 77 (1.1%) |
| **Type 2 diabetes - last 2 years** | 401 (1.6%) | 76 (3.1%) | 325 (1.4%) | 105 (1.7%) | 22 (3.6%) | 83 (1.5%) | 94 (1.5%) | 19 (3.7%) | 113 (1.6%) |
| **Type 2 diabetes - any** | 929 (3.7%) | 202 (8.3%) | 727 (3.2%) | 226 (3.6%) | 50 (8.2%) | 176 (3.1%) | 198 (3.1%) | 40 (7.9%) | 238 (3.4%) |
| **Hyperlipidemia - last 2 years** | 82 (0.3%) | 14 (0.6%) | 68 (0.3%) | 19 (0.3%) | 7 (1.1%) | 12 (0.2%) | 30 (0.5%) | 7 (1.4%) | 37 (0.5%) |
| **Hyperlipidemia - any** | 231 (0.9%) | 39 (1.6%) | 192 (0.9%) | 51 (0.8%) | 13 (2.1%) | 38 (0.7%) | 74 (1.2%) | 15 (3.0%) | 89 (1.3%) |
| **Obesity – last 2 years** | 419 (1.7%) | 57 (2.4%) | 362 (1.6%) | 96 (1.5%) | 17 (2.8%) | 79 (1.4%) | 118 (1.8%) | 16 (3.1%) | 134 (1.9%) |
| **Obesity - any** | 1155 (4.6%) | 150 (6.2%) | 1005 (4.5%) | 291 (4.7%) | 54 (8.8%) | 237 (4.2%) | 440 (6.9%) | 42 (8.3%) | 482 (7.0%) |
| **Tobacco use disorder - last 2 years** | 247 (1.0%) | 28 (1.2%) | 219 (1.0%) | 71 (1.1%) | 11 (1.8%) | 60 (1.1%) | 94 (1.5%) | 14 (2.8%) | 108 (1.6%) |
| **Tobacco use disorder - any** | 501 (2.0%) | 61 (2.5%) | 440 (2.0%) | 140 (2.2%) | 20 (3.3%) | 120 (2.1%) | 248 (3.9%) | 30 (5.9%) | 278 (4.0%) |
| **Dementia – last 2 years** | 146 (0.6%) | 26 (1.1%) | 120 (0.5%) | 32 (0.5%) | 5 (0.8%) | 27 (0.5%) | 52 (0.8%) | 8 (1.6%) | 60 (0.9%) |
| **Dementia - any** | 357 (1.4%) | 72 (3.0%) | 285 (1.3%) | 76 (1.2%) | 15 (2.5%) | 61 (1.1%) | 81 (1.3%) | 10 (2.0%) | 91 (1.3%) |
| **Epilepsy – last 2 years** | 392 (1.6%) | 56 (2.3%) | 336 (1.5%) | 96 (1.5%) | 16 (2.6%) | 80 (1.4%) | 77 (1.2%) | 11 (2.2%) | 88 (1.3%) |
| **Epilepsy - any** | 1267 (5.1%) | 169 (7.0%) | 1098 (4.9%) | 300 (4.8%) | 38 (6.2%) | 262 (4.7%) | 321 (5.0%) | 39 (7.7%) | 360 (5.2%) |
| **Migraine - last 2 years** | 35 (0.1%) | 7 (0.3%) | 28 (0.1%) | 11 (0.2%) | < 5 | 11 (0.2%) | 28 (0.4%) | < 5 | 30 (0.4%) |
| **Migraine - any** | 316 (1.3%) | 58 (2.4%) | 258 (1.1%) | 70 (1.1%) | 7 (1.1%) | 63 (1.1%) | 124 (1.9%) | 8 (1.6%) | 132 (1.9%) |
| **Arthritis/osteoarthritis/joint pain – last 2 years** | 675 (2.7%) | 107 (4.4%) | 568 (2.5%) | 201 (3.2%) | 28 (4.6%) | 173 (3.1%) | 232 (3.6%) | 39 (7.7%) | 271 (3.9%) |
| **Arthritis/osteoarthritis/joint pain - any** | 2496 (10.0%) | 345 (14.2%) | 2151 (9.5%) | 647 (10.4%) | 95 (15.5%) | 552 (9.8%) | 953 (14.9%) | 105 (20.7%) | 1058 (15.3%) |
| **Autoimmune disorders – last 2 years** | 308 (1.2%) | 45 (1.9%) | 263 (1.2%) | 71 (1.1%) | 4 (0.7%) | 67 (1.2%) | 80 (1.2%) | 8 (1.6%) | 88 (1.3%) |
| **Autoimmune disorders - any** | 861 (3.4%) | 117 (4.8%) | 744 (3.3%) | 206 (3.3%) | 22 (3.6%) | 184 (3.3%) | 273 (4.3%) | 28 (5.5%) | 301 (4.4%) |
| **Anxiety - last 2 years** | 1069 (4.3%) | 106 (4.4%) | 963 (4.3%) | 262 (4.2%) | 37 (6.1%) | 225 (4.0%) | 450 (7.0%) | 19 (3.7%) | 469 (6.8%) |
| **Anxiety - any** | 3658 (14.6%) | 421 (17.4%) | 3237 (14.4%) | 890 (14.3%) | 111 (18.2%) | 779 (13.8%) | 1192 (18.6%) | 76 (15.0%) | 1268 (18.3%) |
| **Depressive disorders – last 2 years** | 2119 (8.5%) | 229 (9.5%) | 1890 (8.4%) | 534 (8.6%) | 56 (9.2%) | 478 (8.5%) | 589 (9.2%) | 39 (7.7%) | 628 (9.1%) |
| **Depressive disorders - any** | 6282 (25.1%) | 678 (28.0%) | 5604 (24.8%) | 1534 (24.6%) | 171 (28.0%) | 1363 (24.2%) | 1851 (28.9%) | 145 (28.5%) | 1996 (28.9%) |
| **ADHD - last 2 years** | 221 (0.9%) | 23 (0.9%) | 198 (0.9%) | 43 (0.7%) | 3 (0.5%) | 40 (0.7%) | 174 (2.7%) | 9 (1.8%) | 183 (2.6%) |
| **ADHD - any** | 316 (1.3%) | 27 (1.1%) | 289 (1.3%) | 67 (1.1%) | 5 (0.8%) | 62 (1.1%) | 348 (5.4%) | 14 (2.8%) | 362 (5.2%) |
| **Substance use disorder - last 2 years** | 1868 (7.5%) | 204 (8.4%) | 1664 (7.4%) | 487 (7.8%) | 45 (7.4%) | 442 (7.8%) | 552 (8.6%) | 29 (5.7%) | 581 (8.4%) |
| **Substance use disorder - any** | 5017 (20.1%) | 528 (21.8%) | 4489 (19.9%) | 1274 (20.4%) | 126 (20.6%) | 1148 (20.4%) | 1295 (20.2%) | 82 (16.1%) | 1377 (19.9%) |
| **Sleep disorders - last 2 years** | 99 (0.4%) | 18 (0.7%) | 81 (0.4%) | 29 (0.5%) | 4 (0.7%) | 25 (0.4%) | 52 (0.8%) | < 5 | 56 (0.8%) |
| **Sleep disorders - any** | 278 (1.1%) | 39 (1.6%) | 239 (1.1%) | 76 (1.2%) | 8 (1.3%) | 68 (1.2%) | 124 (1.9%) | 9 (1.8%) | 133 (1.9%) |
| **Alcohol use disorder – last 2 years** | 2280 (9.1%) | 325 (13.4%) | 1955 (8.7%) | 519 (8.3%) | 79 (12.9%) | 440 (7.8%) | 424 (6.6%) | 63 (12.4%) | 487 (7.0%) |
| **Alcohol use disorder - any** | 5986 (24.0%) | 771 (31.8%) | 5215 (23.1%) | 1474 (23.6%) | 195 (31.9%) | 1279 (22.7%) | 1204 (18.8%) | 126 (24.8%) | 1330 (19.2%) |
| **Intellectual disability - last 2 years** | 411 (1.6%) | 47 (1.9%) | 364 (1.6%) | 98 (1.6%) | 10 (1.6%) | 88 (1.6%) | 124 (1.9%) | 8 (1.6%) | 132 (1.9%) |
| **Intellectual disability - any** | 1419 (5.7%) | 158 (6.5%) | 1261 (5.6%) | 317 (5.1%) | 38 (6.2%) | 279 (5.0%) | 310 (4.8%) | 25 (4.9%) | 335 (4.8%) |
| **Pervasive developmental disorder – last 2 years** | 97 (0.4%) | 11 (0.5%) | 86 (0.4%) | 21 (0.3%) | < 5 | 20 (0.4%) | 62 (1.0%) | < 5 | 64 (0.9%) |
| **Pervasive developmental disorder - any** | 226 (0.9%) | 19 (0.8%) | 207 (0.9%) | 52 (0.8%) | < 5 | 50 (0.9%) | 132 (2.1%) | 6 (1.2%) | 138 (2.0%) |
| **Schizophrenia – last 2 years** | 5119 (20.5%) | 481 (19.9%) | 4638 (20.6%) | 1324 (21.2%) | 118 (19.3%) | 1206 (21.4%) | 203 (3.2%) | 15 (3.0%) | 218 (3.2%) |
| **Schizophrenia - any** | 11847 (47.4%) | 1161 (47.9%) | 10686 (47.4%) | 2908 (46.6%) | 308 (50.4%) | 2600 (46.2%) | 429 (6.7%) | 40 (7.9%) | 469 (6.8%) |
| **Bipolar disorder – last 2 years** | 679 (2.7%) | 85 (3.5%) | 594 (2.6%) | 161 (2.6%) | 21 (3.4%) | 140 (2.5%) | 776 (12.1%) | 24 (4.7%) | 800 (11.6%) |
| **Bipolar disorder - any** | 2304 (9.2%) | 297 (12.3%) | 2007 (8.9%) | 552 (8.8%) | 74 (12.1%) | 478 (8.5%) | 1776 (27.7%) | 108 (21.3%) | 1884 (27.2%) |
| **Personality disorders – last 2 years** | 1542 (6.2%) | 164 (6.8%) | 1378 (6.1%) | 357 (5.7%) | 31 (5.1%) | 326 (5.8%) | 420 (6.6%) | 24 (4.7%) | 444 (6.4%) |
| **Personality disorders - any** | 7678 (30.7%) | 807 (33.3%) | 6871 (30.5%) | 1873 (30.0%) | 204 (33.4%) | 1669 (29.6%) | 1563 (24.4%) | 124 (24.4%) | 1687 (24.4%) |
| **Eating disorders – last 2 years** | 112 (0.4%) | 15 (0.6%) | 97 (0.4%) | 18 (0.3%) | < 5 | 17 (0.3%) | 40 (0.6%) | < 5 | 41 (0.6%) |
| **Eating disorders - any** | 370 (1.5%) | 38 (1.6%) | 332 (1.5%) | 90 (1.4%) | 7 (1.1%) | 83 (1.5%) | 155 (2.4%) | 5 (1.0%) | 160 (2.3%) |
| **Nonpsychiatric hospitalizations – last 2 years** | 0.28 (1.00) | 0.51 (1.52) | 0.25 (0.92) | 0.28 (1.05) | 0.50 (1.19) | 0.26 (1.03) | 0.28 (1.07) | 0.56 (1.37) | 0.30 (1.10) |
| **Psychiatric hospitalizations – last 2 years** | 1.16 (2.67) | 1.27 (3.03) | 1.15 (2.62) | 1.16 (2.79) | 1.21 (3.04) | 1.15 (2.76) | 0.94 (2.21) | 0.71 (2.13) | 0.92 (2.21) |
| **Hypertension medication - last 2 years** | 2840 (11.4%) | 552 (22.8%) | 2288 (10.1%) | 675 (10.8%) | 147 (24.1%) | 528 (9.4%) | 613 (9.6%) | 137 (27.0%) | 750 (10.8%) |
| **Hyperlipidemia medication - last 2 years** | 1814 (7.3%) | 296 (12.2%) | 1518 (6.7%) | 405 (6.5%) | 75 (12.3%) | 330 (5.9%) | 483 (7.5%) | 81 (15.9%) | 564 (8.2%) |
| **Smoking cessation medication - last 2 years** | 501 (2.0%) | 89 (3.7%) | 412 (1.8%) | 122 (2.0%) | 26 (4.3%) | 96 (1.7%) | 91 (1.4%) | 20 (3.9%) | 111 (1.6%) |
| **Sleep disorders medication - last 2 years** | 4104 (16.4%) | 455 (18.8%) | 3649 (16.2%) | 1053 (16.9%) | 128 (20.9%) | 925 (16.4%) | 931 (14.5%) | 72 (14.2%) | 1003 (14.5%) |
| **Alzheimer’s disease medication - last 2 years** | 52 (0.2%) | 8 (0.3%) | 44 (0.2%) | 14 (0.2%) | < 5 | 12 (0.2%) | 34 (0.5%) | 7 (1.4%) | 41 (0.6%) |
| **Anxiolytics medication - last 2 years** | 7193 (28.8%) | 879 (36.3%) | 6314 (28.0%) | 1805 (28.9%) | 237 (38.8%) | 1568 (27.8%) | 1137 (17.7%) | 124 (24.4%) | 1261 (18.2%) |
| **Hypnotics sedatives – last 2 years** | 6399 (25.6%) | 764 (31.5%) | 5635 (25.0%) | 1604 (25.7%) | 202 (33.1%) | 1402 (24.9%) | 1190 (18.6%) | 102 (20.1%) | 1292 (18.7%) |
| **Antiepileptics - last 2 years** | 4078 (16.3%) | 468 (19.3%) | 3610 (16.0%) | 1001 (16.0%) | 134 (21.9%) | 867 (15.4%) | 682 (10.6%) | 76 (15.0%) | 758 (11.0%) |
| **Antidepressants - last 2 years** | 11007 (44.1%) | 1194 (49.3%) | 9813 (43.5%) | 2741 (43.9%) | 288 (47.1%) | 2453 (43.5%) | 2518 (39.3%) | 201 (39.6%) | 2719 (39.3%) |
| **Mood stabilizers - last 2 years** | 2697 (10.8%) | 324 (13.4%) | 2373 (10.5%) | 607 (9.7%) | 74 (12.1%) | 533 (9.5%) | 562 (8.8%) | 50 (9.8%) | 612 (8.8%) |
| **Typical antipsychotics - last 2 years** | 8649 (34.6%) | 970 (40.0%) | 7679 (34.0%) | 2085 (33.4%) | 252 (41.2%) | 1833 (32.5%) | 903 (14.1%) | 89 (17.5%) | 992 (14.3%) |
| **Atypical antipsychotics – last 2 years** | 13420 (53.7%) | 1291 (53.3%) | 12129 (53.8%) | 3292 (52.7%) | 341 (55.8%) | 2951 (52.4%) | 2737 (42.7%) | 221 (43.5%) | 2958 (42.8%) |
| **ADHD medication - last 2 years** | 443 (1.8%) | 39 (1.6%) | 404 (1.8%) | 110 (1.8%) | 8 (1.3%) | 102 (1.8%) | 280 (4.4%) | 23 (4.5%) | 303 (4.4%) |
| **Addictive disorders medication - last 2 years** | 2613 (10.5%) | 350 (14.4%) | 2263 (10.0%) | 651 (10.4%) | 95 (15.5%) | 556 (9.9%) | 380 (5.9%) | 56 (11.0%) | 436 (6.3%) |
| **Income negative** | 135 (0.5%) | 10 (0.4%) | 125 (0.6%) | 38 (0.6%) | < 5 | 34 (0.6%) | 56 (0.9%) | < 5 | 57 (0.8%) |
| **Income zero** | 106 (0.4%) | 3 (0.1%) | 103 (0.5%) | 25 (0.4%) | < 5 | 23 (0.4%) | 33 (0.5%) | < 5 | 34 (0.5%) |
| **Income low** | 1340 (5.4%) | 95 (3.9%) | 1245 (5.5%) | 337 (5.4%) | < 5 | 305 (5.4%) | 589 (9.2%) | 30 (5.9%) | 619 (8.9%) |
| **Income medium** | 22455 (89.9%) | 2223 (91.7%) | 20232 (89.7%) | 5605 (89.8%) | < 5 | 5050 (89.7%) | 5502 (85.8%) | 460 (90.6%) | 5962 (86.2%) |
| **Income high** | 944 (3.8%) | 92 (3.8%) | 852 (3.8%) | 239 (3.8%) | < 5 | 221 (3.9%) | 229 (3.6%) | 16 (3.1%) | 245 (3.5%) |
| **Foreign born** | 4625 (18.5%) | 321 (13.2%) | 4304 (19.1%) | 1206 (19.3%) | 86 (14.1%) | 1120 (19.9%) | 1620 (25.3%) | 71 (14.0%) | 1691 (24.4%) |
| **Residence rural** | 4505 (18.0%) | 442 (18.2%) | 4063 (18.0%) | 1160 (18.6%) | 128 (20.9%) | 1032 (18.3%) | 1196 (18.7%) | 116 (22.8%) | 1312 (19.0%) |
| **Civil status alone** | 20248 (81.1%) | 1914 (79.0%) | 18334 (81.3%) | 5031 (80.6%) | 488 (79.9%) | 4543 (80.6%) | 5196 (81.1%) | 399 (78.5%) | 5595 (80.9%) |
| **Having children** | 11252 (45.0%) | 1225 (50.6%) | 10027 (44.5%) | 2832 (45.4%) | 317 (51.9%) | 2515 (44.6%) | 3036 (47.4%) | 298 (58.7%) | 3334 (48.2%) |
| **CVD family history - parent** | 4280 (17.1%) | 353 (14.6%) | 3927 (17.4%) | 1026 (16.4%) | 81 (13.3%) | 945 (16.8%) | 1489 (23.2%) | 92 (18.1%) | 1581 (22.9%) |
| **CVD family history - sibling** | 1882 (7.5%) | 204 (8.4%) | 1678 (7.4%) | 455 (7.3%) | 52 (8.5%) | 403 (7.2%) | 619 (9.7%) | 69 (13.6%) | 688 (9.9%) |
| **Family history of psychiatric disorders** | 9458 (37.9%) | 785 (32.4%) | 8673 (38.4%) | 2369 (37.9%) | 193 (31.6%) | 2176 (38.6%) | 2756 (43.0%) | 164 (32.3%) | 2920 (42.2%) |
| **Parent died before age 60** | 3295 (13.2%) | 298 (12.3%) | 2997 (13.3%) | 831 (13.3%) | 76 (12.4%) | 755 (13.4%) | 781 (12.2%) | 65 (12.8%) | 846 (12.2%) |

***Educational attainment was excluded as there were more than 30% missing values in the Danish cohort.**

**Supplementary Table 6.** Characteristics of the study population in the derivation and testing data sets regarding cardiovascular outcomes.

**A) Swedish Cohort**

|  | **Training data**  N= 38,999  N (%) | **Testing data**  N= 9,750  N (%) |
| --- | --- | --- |
| **Incident CVD: total** | **3,663** | **915** |
| **Incident diagnosis from the NPR** | **2,822 (77%)** | **700 (76.4%)** |
| Ischemic heart disease | 442 (12%) | 106 (11.6%) |
| Cerebrovascular diseases and transient ischemic attack | 806 (22%) | 179 (19.5%) |
| Thromboembolic diseases | 545 (14.9%) | 134 (14.6%) |
| Heart failure | 357 (9.7%) | 93 (10.1%) |
| Arteriosclerosis | 141 (3.8%) | 36 (3.9%) |
| Arrhythmias | 531 (14.5%) | 152 (16.6%) |
| **Incident medication prescription from the PDR** | **487 (13.3%)** | **110 (12%)** |
| **CVD as a cause of death from the CDR** | **354 (9.6%)** | **105 (11.5%)** |

**B) Danish Cohort**

|  | **Training data**  N=22,557  N (%) | **Testing data (internal)**  N=6244  N (%) | **Testing data (temporal)**  N=6917  N (%) |
| --- | --- | --- | --- |
| **Incident CVD: total** | **2423** | **609** | **508** |
| **Incident diagnosis from the NPR** | **1844 (76.1%)** | **464 (76.1%)** | **395 (77.7%)** |
| Ischemic heart disease | 347 (14.3%) | 87 (14.2%) | 56 (11.0%) |
| Cerebrovascular diseases and transient ischemic attack | 510 (21.0%) | 128 (20.9%) | 105 (20.7%) |
| Thromboembolic diseases | 400 (16.5%) | 101 (16.5%) | 82 (16.1%) |
| Heart failure - broad | 25 (1.0%) | 6 (1.0%) | 6 (1.2%) |
| Arteriosclerosis | 151 (6.2%) | 38 (6.2%) | 31 (6.1%) |
| Arrhythmias | 411 (17.0%) | 104 (17.0%) | 115 (22.6%) |
| **Incident medication prescription from the PDR** | **387 (16.0%)** | **98 (16.0%)** | **69 (13.6%)** |
| **CVD as a cause of death from the CDR** | **192 (7.9%)** | **49 (8.0%)** | **44 (8.7%)** |

**Supplementary Table 7.** Hyperparameter ranges and optimal hyperparameters.

|  | Searched range | Optimal Denmark | Optimal Sweden |
| --- | --- | --- | --- |
| Elastic net |  |  |  |
| - penalty | [None, ‘l1’,’l2’,’elastic_net’] | ‘l1’ | ‘l1’ |
| - l1_ratio | [0.1,0.2,0.3,0.4,0.5,0.6,0.7,0.8,0.9] | None | None |
| - C | [0.1,0.2,0.3,0.4,0.5,0.6,0.7,0.8,0.9] | 0.4 | 0.5 |
| XGBoost |  |  |  |
| - colsample_bytree | [0.3, 0.4, 0.5, 0.6, 0.7, 0.8, 0.9,1.0] | 0.5 | 0.6 |
| - learning_rate | [0.01,0.05,0.1,0.5] | 0.1 | 0.5 |
| - max_depth | [1,2,3,4,5,6] | 2 | 2 |
| - n_estimators | [10,50,100,150,200] | 100 | 150 |

**Supplementary Table 8.** Variables and corresponding parameter estimates in lasso penalized logistic regression on the Swedish (A) and Danish (B) model.

A)

| **Predictor** | **Coefficient** |
| --- | --- |
| age | 0.813508 |
| obesity any | 0.302111 |
| hypertension med 2 years | 0.281447 |
| hypertension any | 0.281298 |
| diabetes2 any | 0.273276 |
| sex male | 0.256152 |
| epilepsy any | 0.25322 |
| diabetes1 any | 0.221364 |
| intellectual disability any | 0.21948 |
| cvd sibling 1 | 0.198492 |
| mood stabilizers med 2 years | 0.183324 |
| alcohol any | 0.160585 |
| smoking cessation med 2 years | 0.15431 |
| antiepileptics med 2 years | 0.131673 |
| anxiolytics med 2 years | 0.11657 |
| substance use any | 0.115374 |
| arthritis pain any | 0.100731 |
| tobacco any | 0.100003 |
| cvd parent 1 | 0.094389 |
| children | 0.094124 |
| schizophrenia 2 years | -0.08986 |
| antipsychotics typical med 2 years | 0.071444 |
| antidepressants med 2 years | 0.070071 |
| income medium | 0.066024 |
| hyperlipidemia med 2 years | 0.06284 |
| schizophrenia any | 0.062368 |
| hypnotics sedatives med 2 years | 0.049539 |
| cvd parent missing | 0.044956 |
| dead parent missing | 0.044956 |
| n psych hospitalizations 2 years | 0.040192 |
| personality any | 0.033851 |
| antipsychotics atypical med 2 years | -0.0293 |
| n hospitalizations 2 years | 0.025138 |
| psych family 1 | 0.021709 |
| income low | -0.02086 |
| personality 2 years | 0.018467 |
| bipolar any | 0.016009 |
| attention deficit 2 years | 0.014988 |
| dead parent 1 | 0.012012 |
| diabetes1 2 years | 0.004226 |
| foreign born | 0.002001 |
| sleep disorder med 2 years | 0.001604 |
| depressive any | -0.00072 |
| depressive 2 years | -0.00043 |

B)

| **Predictor** | **Coefficient** |
| --- | --- |
| hypertension any | 0.666333585 |
| diabetes2 any | 0.56796745 |
| diabetes2 2 years | 0.514573521 |
| migraine any | 0.405695519 |
| income high | -0.394354738 |
| eating any | 0.366113612 |
| obesity any | 0.326117541 |
| arthritis pain any | 0.318593297 |
| age | 0.311948569 |
| hypnotics sedatives med 2 years | 0.285675912 |
| alcohol 2 years | 0.285033498 |
| sleep disorder med 2 years | -0.260695057 |
| cvd sibling 1 | 0.235798413 |
| ADHD med 2 years | 0.213881267 |
| substance use 2 years | 0.210682043 |
| hypertension med 2 years | 0.197210557 |
| civil status alone | -0.18873518 |
| cvd parent 1 | 0.186029422 |
| diabetes1 any | 0.167541995 |
| dementia any | 0.165155137 |
| eating 2 years | 0.1648578 |
| intellectual disability 2 years | 0.160353601 |
| anxiety any | 0.14932496 |
| addictive disorders med 2 years | 0.134186084 |
| autoimmune any | 0.128239416 |
| epilepsy any | 0.127694118 |
| hyperlipidemia med 2 years | 0.123096154 |
| smoking cessation med 2 years | 0.122374891 |
| anxiolytics med 2 years | 0.118624781 |
| depressive 2 years | -0.11509971 |
| dead parent 1 | 0.104247398 |
| substance use any | 0.103347132 |
| alcohol any | 0.100074478 |
| sex male | 0.097170258 |
| depressive any | 0.094535838 |
| psych family 1 | 0.088197176 |
| n hospitalizations 2 years | 0.08349278 |
| schizophrenia 2 years | 0.081776827 |
| sleep any | 0.077117839 |
| intellectual disability any | 0.067763621 |
| epilepsy 2 years | -0.060520533 |
| personality any | -0.050672688 |
| hyperlipidemia 2 years | 0.050247754 |
| schizophrenia any | -0.047647553 |
| tobacco any | -0.046318934 |
| children | 0.042025327 |
| personality 2 years | 0.037406118 |
| antidepressants med 2 years | 0.037393792 |
| n psych hospitalizations 2 years | 0.031033931 |
| antiepileptics med 2 years | 0.021331375 |
| antipsychotics atypical med 2 years | 0.019786184 |
| antipsychotics typical med 2 years | 0.013972483 |
| anxiety 2 years | 0.01139314 |
| cvd parent missing | -0.010233889 |
| cvd sibling missing | -0.010233889 |
| dead parent missing | -0.010233889 |
| psych family missing | -0.010233889 |
| residence rural | 0.009148513 |

**Supplementary Table 9.** Additional performance measures on the internal hold-out test sets for Sweden (A) and Denmark (B) for risk thresholds at 7.5%, 10%, and 20%.

A)

| Risk threshold | 7.5% | 10% | 20% |
| --- | --- | --- | --- |
| F1-score | 0.270 (0.268, 0.273) | 0.299 (0.296, 0.304) | 0.279 (0.276, 0.283) |
| MCC | 0.273 (0.269, 0.277) | 0.276 (0.272, 0.279) | 0.201 (0.197, 0.205) |
| Brier | 0.079 (0.078, 0.080) | 0.079 (0.078, 0.080) | 0.079 (0.078, 0.080) |

B)

| Risk threshold | 7.5% | 10% | 20% |
| --- | --- | --- | --- |
| F1-score | 0.263 (0.260, 0.267) | 0.275 (0.271, 0.278) | 0.267 (0.263, 0.272) |
| MCC | 0.245 (0.243, 0.247) | 0.222 (0.218, 0.226) | 0.197 (0.192, 0.202) |
| Brier | 0.083 (0.082, 0.083) | 0.083 (0.082, 0.083) | 0.083 (0.082, 0.083) |

**Supplementary Table 10.** Performance measures on the temporal hold-out test set on the Danish data for risk threshold at 10%.

| Risk threshold | 7.5% | 10% | 20% |
| --- | --- | --- | --- |
| Sensitivity | 0.760 (0.754, 0.764) | 0.657 (0.651, 0.665) | 0.331 (0.327, 0.335) |
| Specificity | 0.627 (0.621, 0.633) | 0.736 (0.732, 0.740) | 0.900 (0.890, 0.909) |
| Positive predictive value | 0.139 (0.134, 0.143) | 0.165 (0.160, 0.171) | 0.208 (0.201, 0.214) |
| Negative predictive value | 0.971 (0.968, 0.974) | 0.964 (0.961, 0.968) | 0.944 (0.940, 0.948) |
| F1-score | 0.235 (0.231, 0.239) | 0.263 (0.258, 0.270) | 0.256 (0.251, 0.262) |
| MCC | 0.256 (0.252, 0.262) | 0.257 (0.270, 0.263) | 0.192 (0.188, 0.195) |
| Brier | 0.066 (0.065, 0.068) | 0.066 (0.065, 0.068) | 0.066 (0.065, 0.068) |
| True positives | 386 | 334 | 168 |
| True negatives | 4020 | 4715 | 5770 |
| False positives | 2389 | 1694 | 639 |
| False negatives | 122 | 174 | 340 |

**Supplementary Table 11.** Additional performance measures on external validation on Swedish (A) and Danish (B) cohort for risk thresholds at 7.5%, 10%, and 20%.

A)

| Risk threshold | 7.5% | 10% | 20% |
| --- | --- | --- | --- |
| Sensitivity | 0.805 (0.802, 0.810) | 0.642 (0.639, 0.647) | 0.173 (0.170, 0.177) |
| Specificity | 0.565 (0.563, 0.568) | 0.713 (0.711, 0.716) | 0.953 (0.951, 0.956) |
| Positive predictive value | 0.161 (0.159, 0.164) | 0.188 (0.186, 0.192) | 0.278 (0.276, 0.284) |
| Negative predictive value | 0.966 (0.963, 0.968) | 0.950 (0.948, 0.953) | 0.918 (0.915, 0.920) |
| F1-score | 0.268 (0.262, 0.272) | 0.291 (0.288, 0.295) | 0.213 (0.210, 0.218) |
| MCC | 0.284 (0.281, 0.288) | 0.257 (0.254, 0.261) | 0.156 (0.152, 0.160) |
| Brier | 0.080 (0.079, 0.080) | 0.080 (0.079, 0.080) | 0.080 (0.079, 0.080) |
| True positives | 737 | 587 | 158 |
| True negatives | 4994 | 6298 | 8424 |
| False positives | 3841 | 2537 | 411 |
| False negatives | 178 | 328 | 757 |

B)

| Risk threshold | 7.5% | 10% | 20% |
| --- | --- | --- | --- |
| Sensitivity | 0.870 (0.867, 0.874) | 0.608 (0.603, 0.613) | 0.491 (0.488, 0.495) |
| Specificity | 0.388 (0.384, 0.389) | 0.696 (0.692, 0.698) | 0.791 (0.787, 0.792) |
| Positive predictive value | 0.133 (0.130, 0.135) | 0.178 (0.175, 0.181) | 0.202 (0.199, 0.204) |
| Negative predictive value | 0.965 (0.962, 0.967) | 0.943 (0.940, 0.945) | 0.935 (0.932, 0.937) |
| F1-score | 0.231 (0.228, 0.233) | 0.275 (0.271, 0.278) | 0.287 (0.284, 0.289) |
| MCC | 0.252 (0.248, 0.255) | 0.222 (0.218, 0.226) | 0.214 (0.210, 0.219) |
| Brier | 0.083 (0.082, 0.084) | 0.083 (0.082, 0.084) | 0.083 (0.082, 0.084) |
| True positives | 530 | 370 | 299 |
| True negatives | 2186 | 3922 | 4457 |
| False positives | 3450 | 1714 | 1179 |
| False negatives | 79 | 239 | 310 |

**Supplementary Table 12.** Performance measures on the internal hold-out test sets for Sweden (A) and Denmark (B) for risk thresholds at 10% separated by sex.

A)

|  | Male | | Female |  |
| --- | --- | --- | --- | --- |
| Risk group | High | Low | High | Low |
| 5-year event rate with 95% CI | 0.252 (0.217, 0.291) | 0.076 (0.069, 0.085) | 0.251 (0.216, 0.290) | 0.073 (0.065, 0.081) |
| Odds ratio with 95% CI unadjusted | 4.08 (3.25, 5.11) | | 4.29 (3.41, 5.39) | |
| Adjusted for age | 0.74 (0.52 - 1.04) | | 1.09 (0.78 - 1.51) | |
| AUC | 0.741 (0.738, 0.747) | | 0.750 (0.745, 0.756) | |
| AUPRC | 0.223 (0.218, 0.229) | | 0.234 (0.228, 0.239) | |
| Sensitivity | 0.629 (0.623, 0.632) | | 0.731 (0.726, 0.736) | |
| Specificity | 0.715 (0.713, 0.718) | | 0.687 (0.683, 0.689) | |
| Positive predictive value | 0.184 (0.181, 0.187) | | 0.197 (0.193, 0.199) | |
| Negative predictive value | 0.950 (0.947, 0.952) | | 0.960 (0.958, 0.963) | |
| F1-score | 0.285 (0.281, 0.288) | | 0.309 (0.305, 0.313) | |
| MCC | 0.247 (0.242, 0.251) | | 0.302 (0.328, 0.307) | |
| Brier | 0.079 (0.077, 0.080) | | 0.079 (0.078, 0.079) | |
| True positives | 300 | | 320 | |
| True negatives | 3341 | | 2859 | |
| False positives | 1330 | | 1305 | |
| False negatives | 177 | | 118 | |

B)

|  | Male | | Female |  |
| --- | --- | --- | --- | --- |
| Risk group | High | Low | High | Low |
| 5-year event rate with 95% CI | 0.266 [0.243, 0.310] | 0.079 [0.074, 0.088] | 0.264 [0.237, 0.307] | 0.076 [0.069, 0.083] |
| Odds ratio with 95% CI unadjusted | 4.16 [3.51 - 4.88] | | 4.33 [3.79 – 5.13] | |
| Adjusted for age | 0.78 [0.60 - 1.01] | | 0.13 [0.81- 1.33] | |
| AUC | 0.727 (0.722, 0.734) | | 0.719 (0.716, 0.724) | |
| AUPRC | 0.226 (0.223, 0.230) | | 0.224 (0.221, 0.228) | |
| Sensitivity | 0.621 (0.619, 0.627) | | 0.639 (0.635, 0.643) | |
| Specificity | 0.719 (0.716, 0.722) | | 0.667 (0.663, 0.669) | |
| Positive predictive value | 0.188 (0.185, 0.193) | | 0.177 (0.173, 0.181) | |
| Negative predictive value | 0.948 (0.945, 0.951) | | 0.942 (0.940, 0.945) | |
| F1-score | 0.290 (0.286, 0.294) | | 0.277 (0.272, 0.281) | |
| MCC | 0.248 (0.243, 0.253) | | 0.227 (0.222, 0.233) | |
| Brier | 0.080 (0.080, 0.081) | | 0.085 (0.085, 0.086) | |
| True positives | 208 | | 175 | |
| True negatives | 2304 | | 1621 | |
| False positives | 900 | | 811 | |
| False negatives | 127 | | 99 | |

**Supplementary Table 13.** Performance measures on the internal hold-out test sets for Sweden (A) and Denmark (B) for risk thresholds at 10% separated by age.

A)

|  | Age < 50 | | Age => 50 | |
| --- | --- | --- | --- | --- |
| Risk group | High | Low | High | Low |
| 5-year event rate with 95% CI | 0.075 (0.056, 0.099) | 0.036 (0.031, 0.042) | 0.260 (0.223, 0.300) | 0.148 (0.137, 0.159) |
| Odds ratio with 95% CI unadjusted | 2.16 (1.52, 3.08) | | 2.03 (1.63, 2.52) | |
| Adjusted for age and sex | 1.17 (0.77, 1.80) | | 0.81 (0.59, 1.11) | |
| AUC | 0.746 (0.740, 0.751) | | 0.744 (0.739, 0.749) | |
| AUPRC | 0.233 (0.229, 0.235) | | 0.235 (0.231, 0.238) | |
| Sensitivity | 0.667(0.659, 0.674) | | 0.689 (0.693, 0.694) | |
| Specificity | 0.711 (0.703, 0.719) | | 0.691 (0.682, 0.696) | |
| Positive predictive value | 0.192 (0.186, 0.195) | | 0.188 (0.182,0.94) | |
| Negative predictive value | 0.954 (0.948, 0.961) | | 0.955 (0.949,0.960) | |
| F1-score | 0.299 (0.309, 0.315) | | 0.296 (0.310, 0.317) | |
| MCC | 0.251 (0.247, 0.256) | | 0.263 (0.258, 0.268) | |
| Brier | 0.079 (0.078, 0.080) | | 0.073 (0.071, 0.075) | |
| True positives | 317 | | 303 | |
| True negatives | 3275 | | 2925 | |
| False positives | 1330 | | 1305 | |
| False negatives | 158 | | 137 | |

B)

|  | Age < 50 | | Age => 50 | |
| --- | --- | --- | --- | --- |
| Risk group | High | Low | High | Low |
| 5-year event rate with 95% CI | 0.081 [0.058, 0.102] | 0.039 [0.032, 0.043] | 0.273 [0.232, 0.309] | 0.150 [0.139, 0.161] |
| Odds ratio with 95% CI unadjusted | 2.23 [1.67 - 2.98] | | 4.21 [3.67 - 4.98] | |
| Adjusted for age and sex | 1.19 [0.75 - 1.73] | | 0.86 [0.63 - 1.15] | |
| AUC | 0.710 (0.702, 0.719) | | 0.734 (0.726, 0.741) | |
| AUPRC | 0.230 (0.225, 0.234) | | 0.215 (0.209, 0.220) | |
| Sensitivity | 0.630 (0.627, 0.638) | | 0.460 (0.453, 0.470) | |
| Specificity | 0.601 (0.698, 0.605) | | 0.883 (0.880, 0.886) | |
| Positive predictive value | 0.176 (0.173, 0.180) | | 0.221 (0.216, 0.226) | |
| Negative predictive value | 0.923 (0.921, 0.927) | | 0.958 (0.955, 0.960) | |
| F1-score | 0.275 (0.272, 0.280) | | 0.299 (0.293, 0.304) | |
| MCC | 0.190 (0.185, 0.195) | | 0.258 (0.254, 0.264) | |
| Brier | 0.060 (0.057, 0.062) | | 0.058 (0.057, 0.059) | |
| True positives | 273 | | 81 | |
| True negatives | 1930 | | 2142 | |
| False positives | 1279 | | 284 | |
| False negatives | 160 | | 95 | |

**Supplementary Figure 1.** Internal test AUC curve for Swedish (A) and Danish (B) cohort.

A)


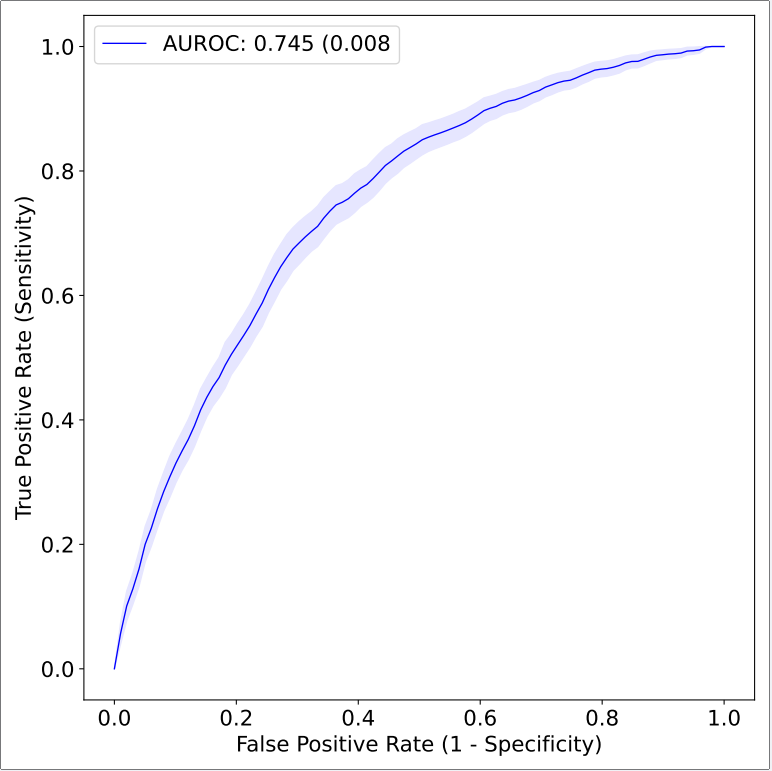


B)

***
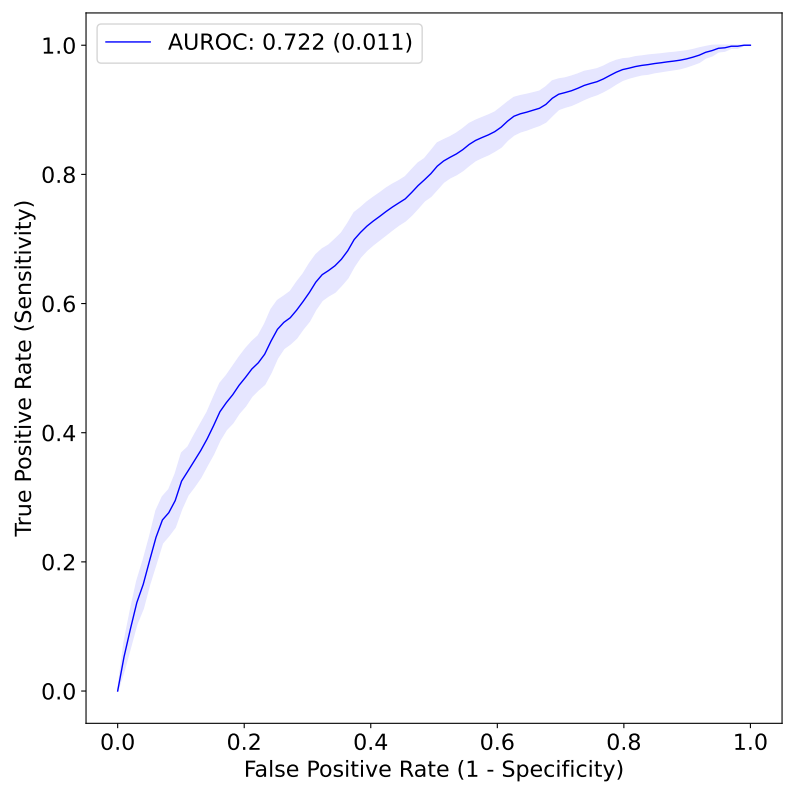
***

**Supplementary Figure 2.** Internal test AUPRC curve for Swedish (A) and Danish (B) cohort.

A)


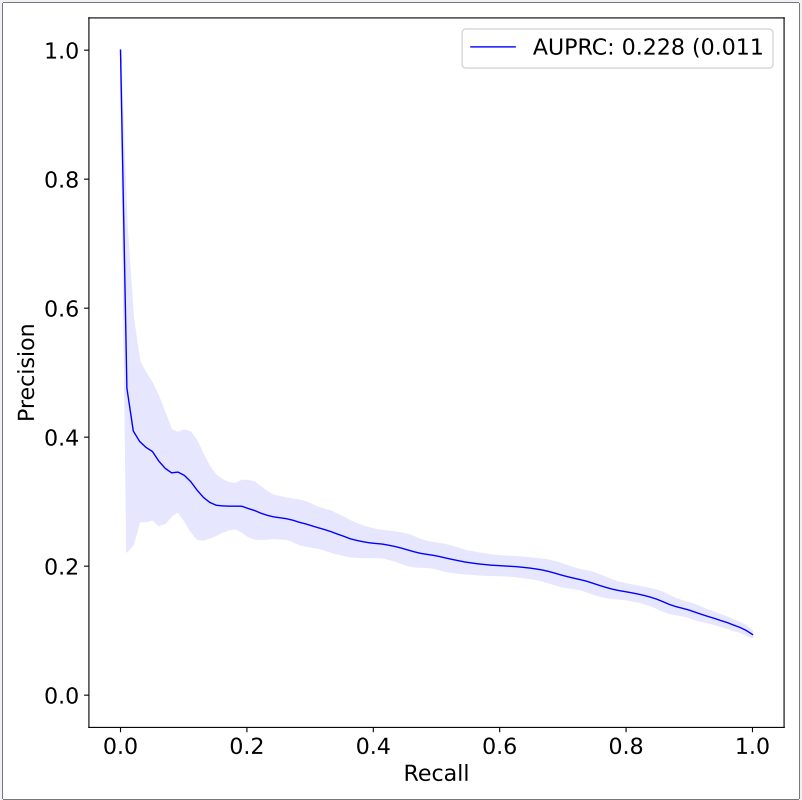


B)


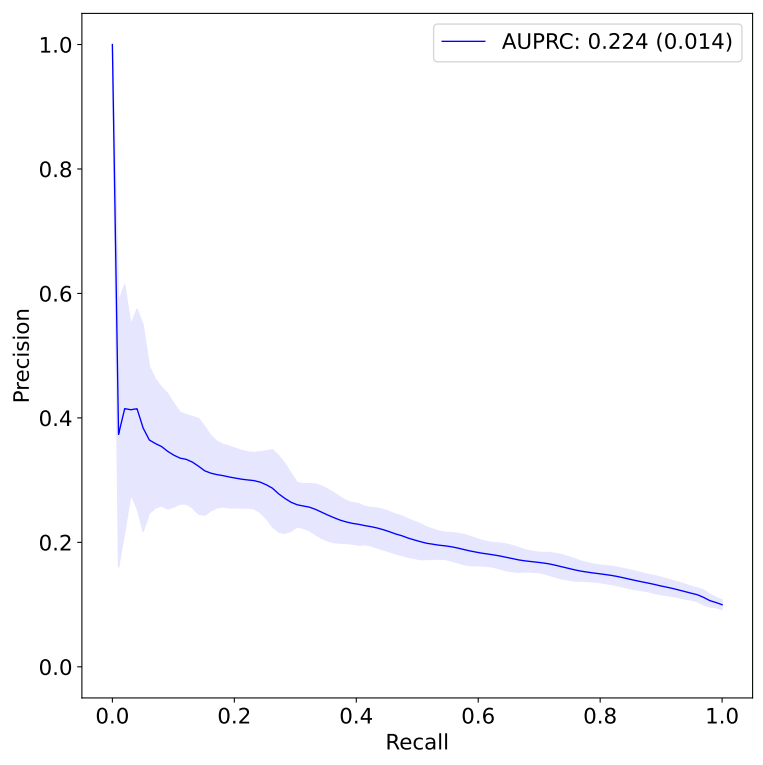


**Supplementary Figure 3.** Internal test calibration plot for Swedish (A) and Danish (B) cohort.

A)


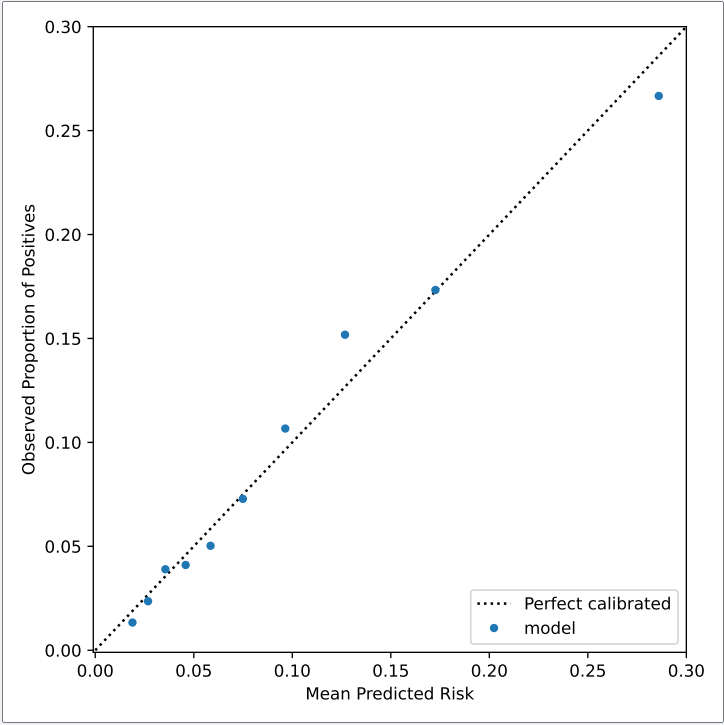


B)


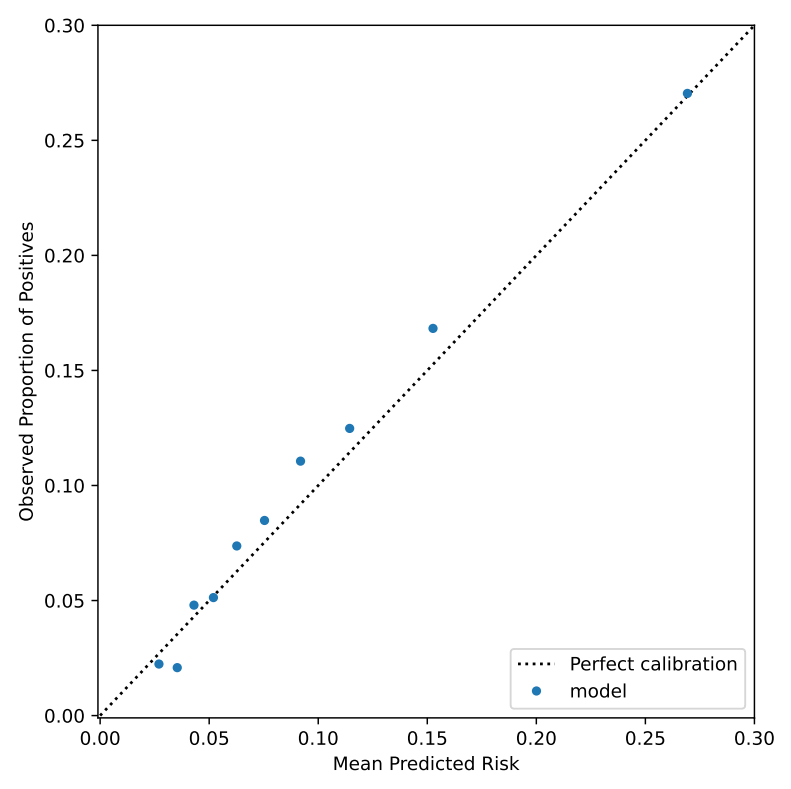


**Supplementary Figure 4.** Test AUC curve for temporal hold-out test set Danish cohort.

***
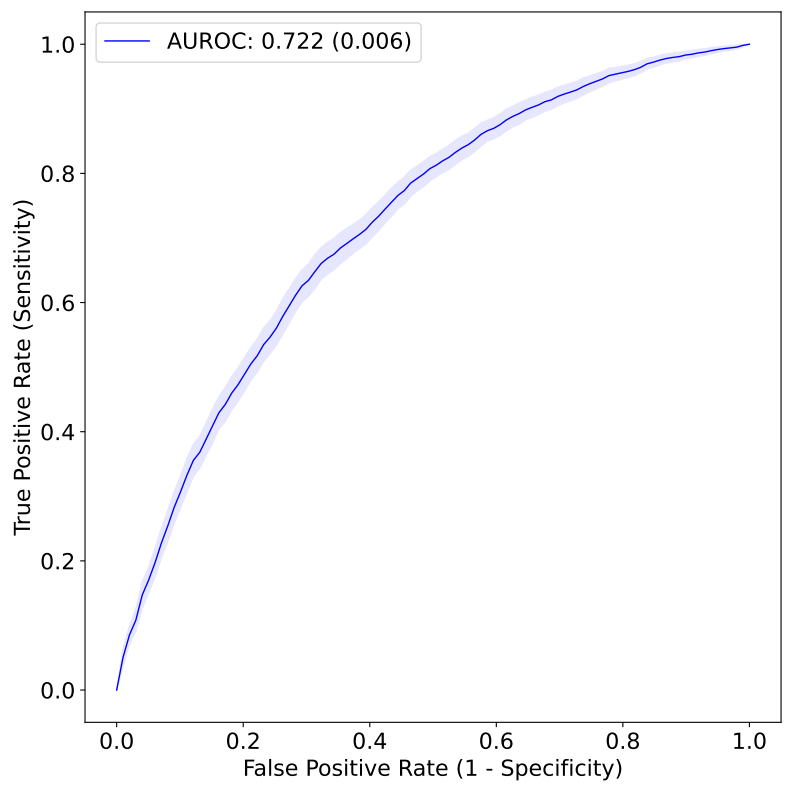
***

**Supplementary Figure 5.** Test AUPRC curve for temporal hold-out test set Danish cohort.


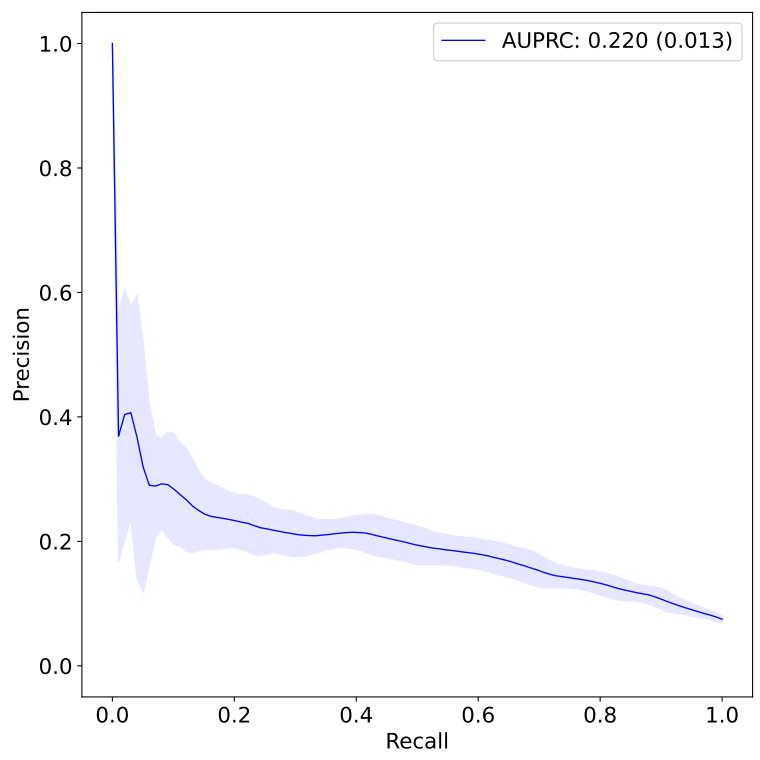


**Supplementary Figure 6.** Test calibration plot for temporal hold-out test set Danish cohort.

***
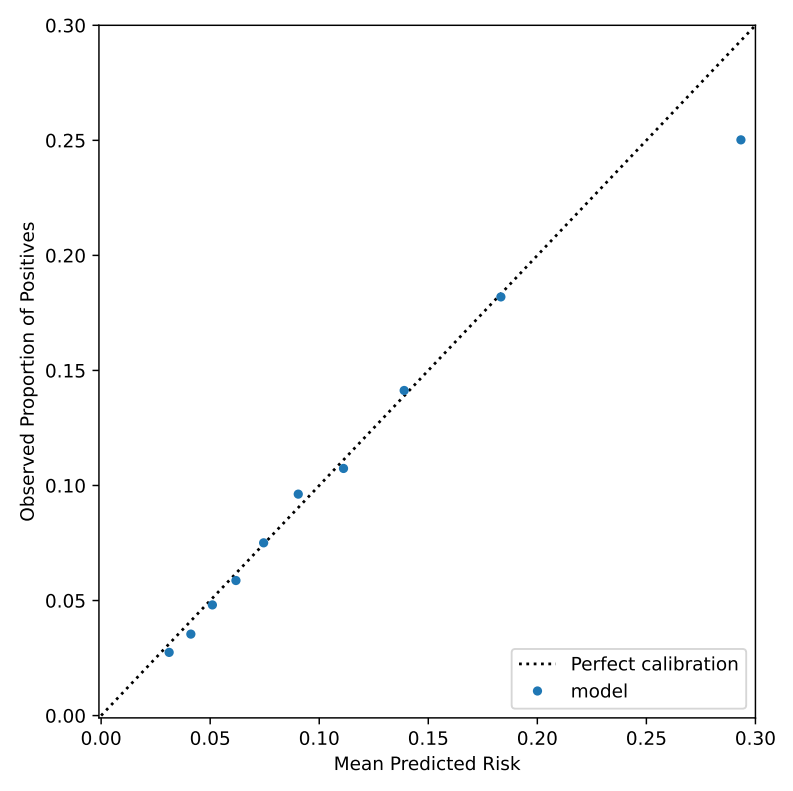
***

**Supplementary Figure 7.** Mean SHAP values for Swedish (A) and Danish (B) cohort.

A)

**
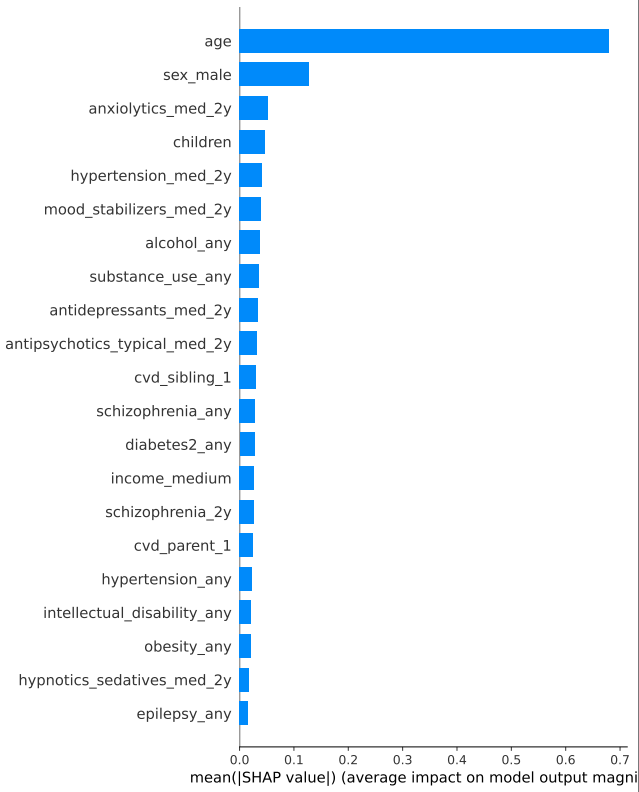
**

**B)**

**
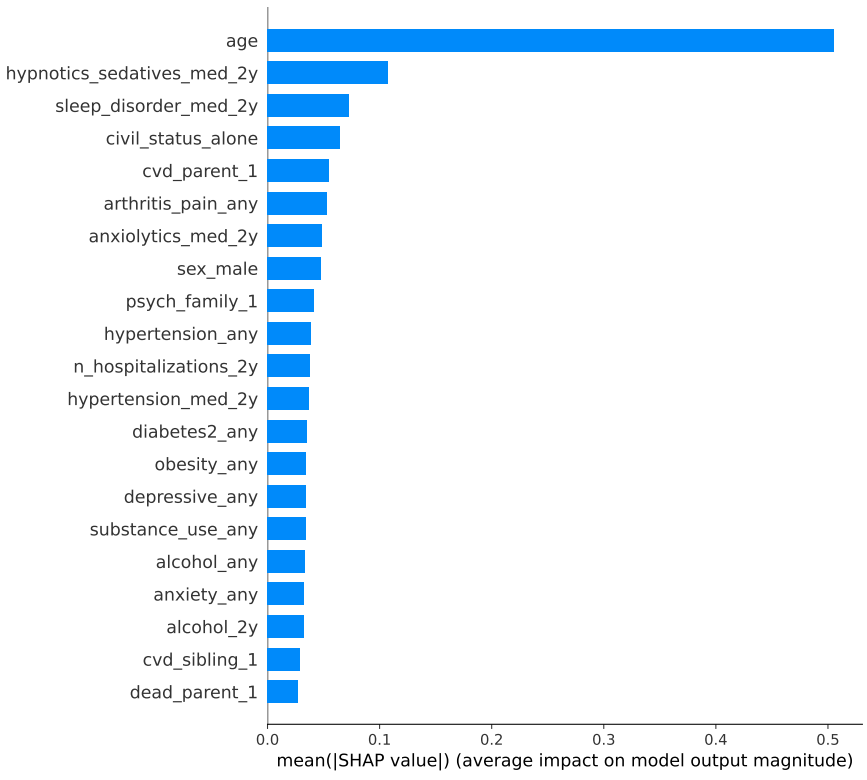
**

**Supplementary Figure 8.** External test AUC curve for Swedish (A) and Danish (B) cohort.

A)


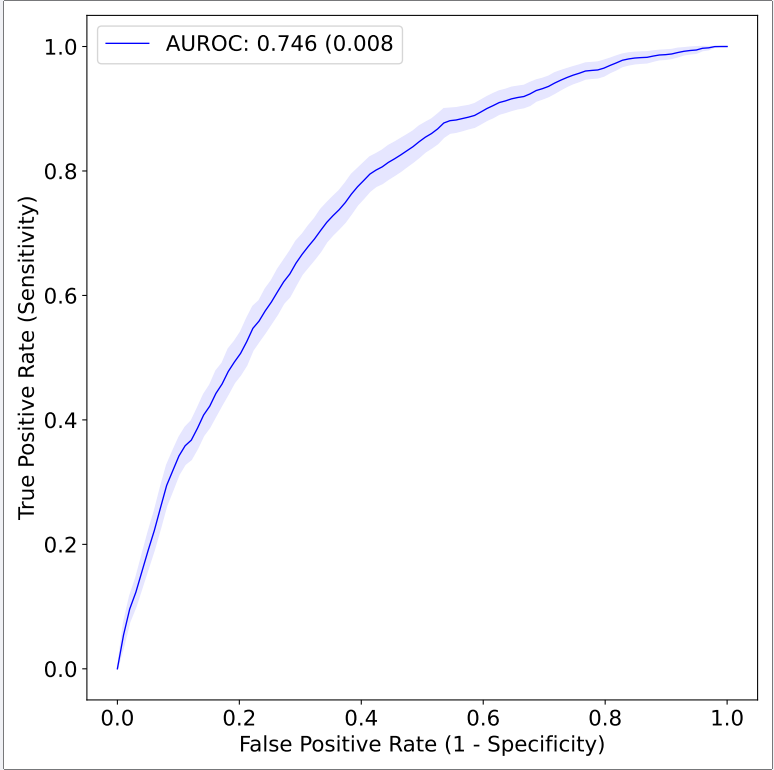


B)

***
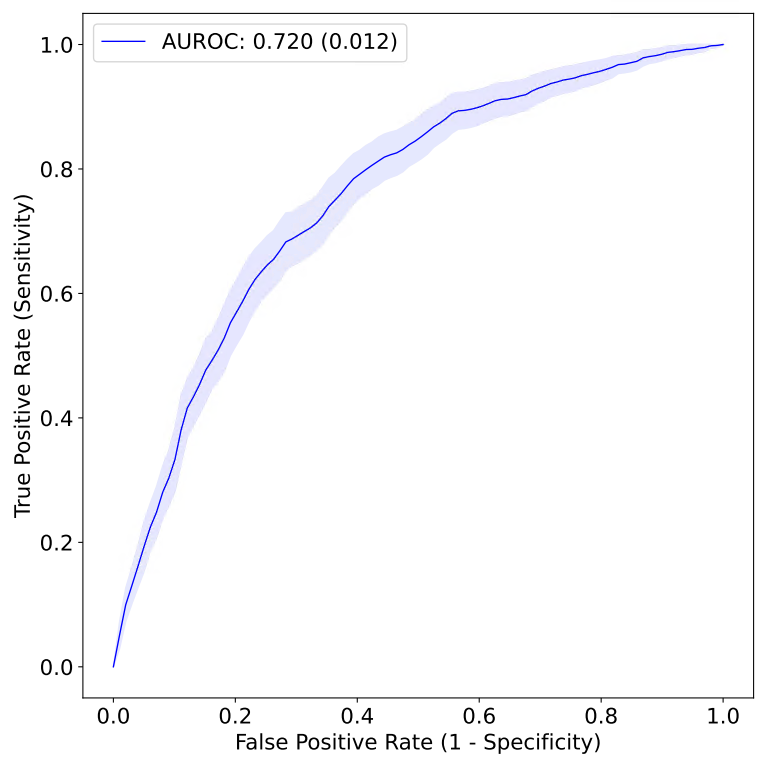
***

**Supplementary Figure 9.** External test AUPRC curve for Swedish (A) and Danish (B) cohort.

A)


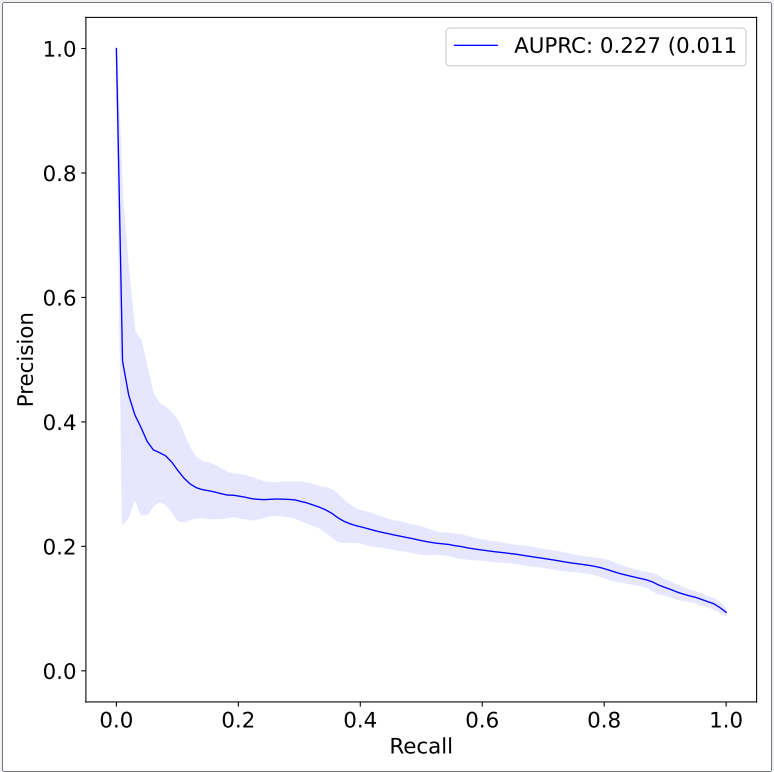


B)


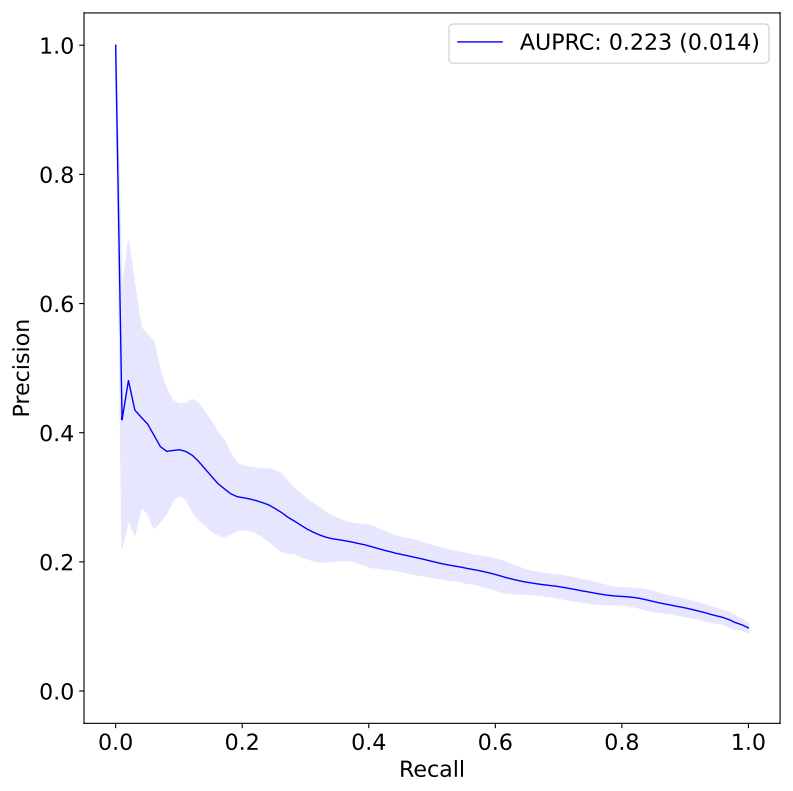


**Supplementary Figure 10.** External test calibration plot for Swedish (A) and Danish (B) cohort.

A)


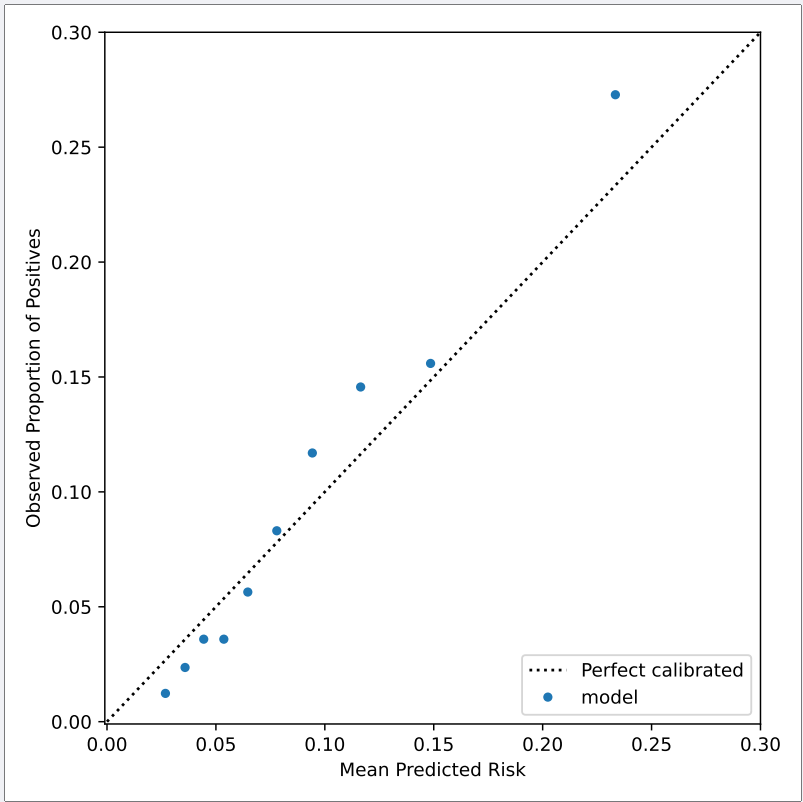


B)

***
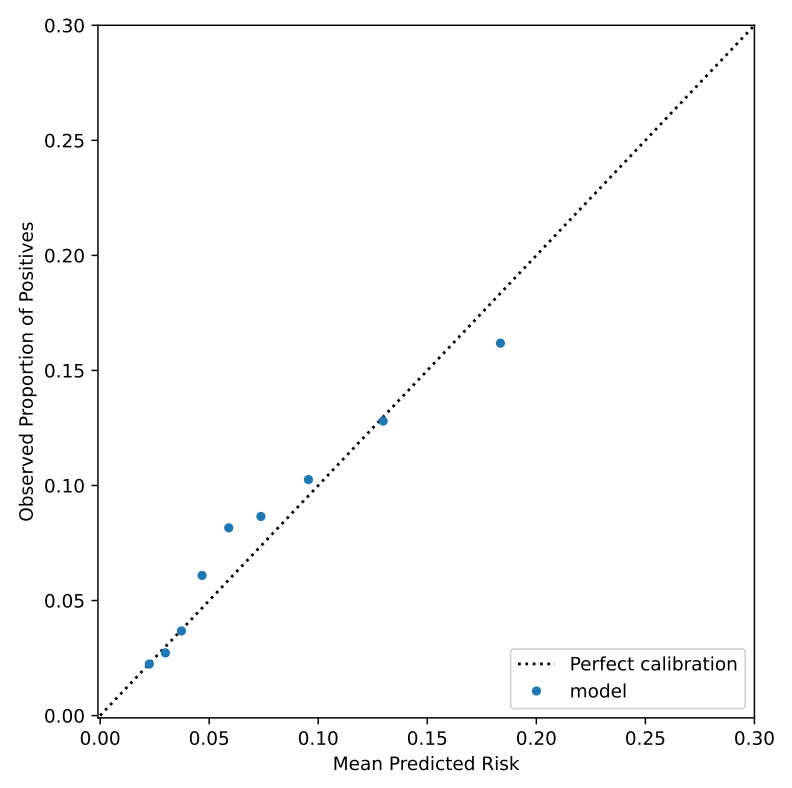
***

**Supplementary Figure 11.** Internal test calibration plot for Swedish (A) and Danish (B) cohort separated by age.

A)


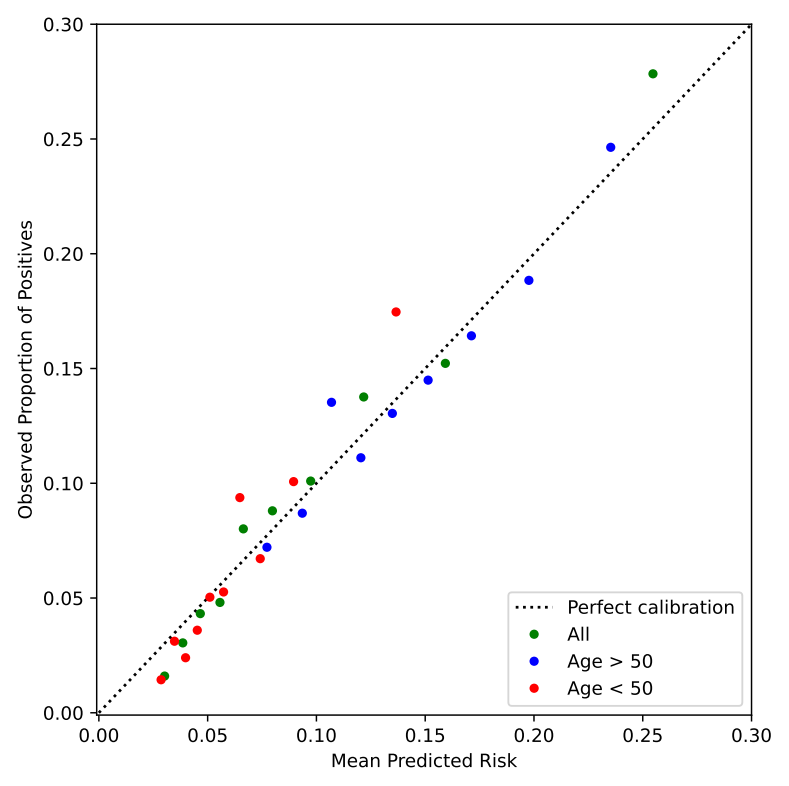


B)


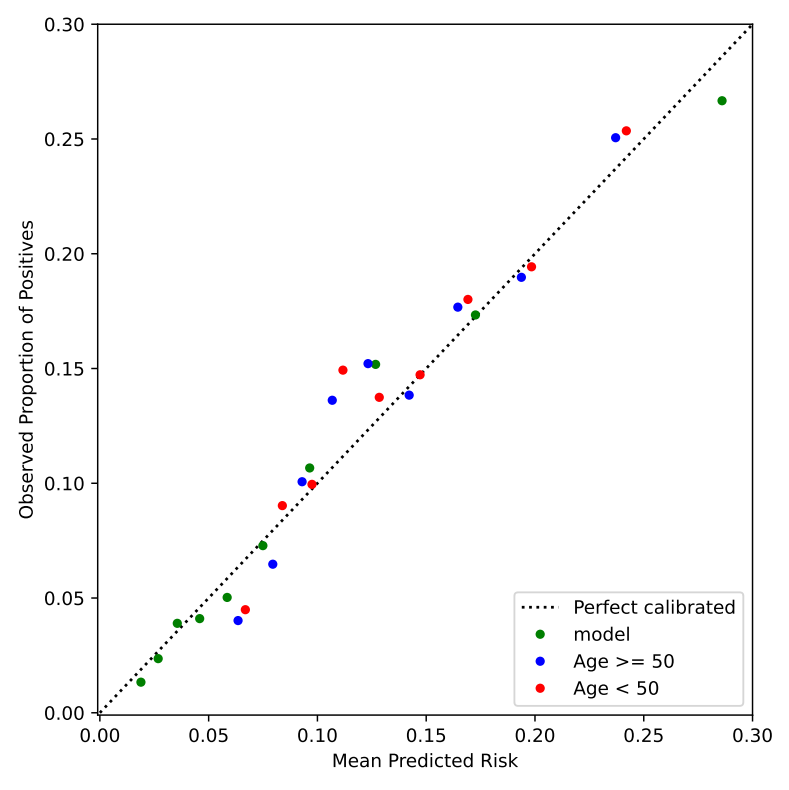

Supplement: online supplemental file 1 [file bmjment-29-1-s001.docx]
